# Interspecies Isobaric Labeling-Based Quantitative Proteomics Reveals Protein Changes in the Ovary of *Aedes aegypti* Coinfected With ZIKV and *Wolbachia*

**DOI:** 10.3389/fcimb.2022.900608

**Published:** 2022-07-07

**Authors:** Luís Felipe Costa Ramos, Michele Martins, Jimmy Rodriguez Murillo, Gilberto Barbosa Domont, Danielle Maria Perpétua de Oliveira, Fábio César Sousa Nogueira, Rafael Maciel-de-Freitas, Magno Junqueira

**Affiliations:** ^1^ Departamento de Bioquímica, Instituto de Química, Universidade Federal do Rio de Janeiro, Rio de Janeiro, Brazil; ^2^ Division of Chemistry I, Department of Medical Biochemistry and Biophysics, Karolinska Institute, Stockholm, Sweden; ^3^ Laboratório de Mosquitos Transmissores de Hematozoários, Instituto Oswaldo Cruz, Fiocruz, Rio de Janeiro, Brazil; ^4^ Department of Arbovirology, Bernhard-Nocht-Institute for Tropical Medicine, Hamburg, Germany

**Keywords:** proteome, quantitative, *Aedes aegypti*, *Wolbachia*, Zika virus, juvenile hormone, immune defense

## Abstract

Zika is a vector-borne disease caused by an arbovirus (ZIKV) and overwhelmingly transmitted by *Ae. aegypti*. This disease is linked to adverse fetal outcomes, mostly microcephaly in newborns, and other clinical aspects such as acute febrile illness and neurologic complications, for example, Guillain-Barré syndrome. One of the most promising strategies to mitigate arbovirus transmission involves releasing *Ae. aegypti* mosquitoes carrying the maternally inherited endosymbiont bacteria *Wolbachia pipientis*. The presence of *Wolbachia* is associated with a reduced susceptibility to arboviruses and a fitness cost in mosquito life-history traits such as fecundity and fertility. However, the mechanisms by which *Wolbachia* influences metabolic pathways leading to differences in egg production remains poorly known. To investigate the impact of coinfections on the reproductive tract of the mosquito, we applied an isobaric labeling-based quantitative proteomic strategy to investigate the influence of *Wolbachia w*Mel and ZIKV infection in *Ae. aegypti* ovaries. To the best of our knowledge, this is the most complete proteome of *Ae. aegypti* ovaries reported so far, with a total of 3913 proteins identified, were also able to quantify 1044 *Wolbachia* proteins in complex sample tissue of *Ae. aegypti* ovary. Furthermore, from a total of 480 mosquito proteins modulated in our study, we discuss proteins and pathways altered in *Ae. aegypti* during ZIKV infections, *Wolbachia* infections, coinfection *Wolbachia*/ZIKV, and compared with no infection, focusing on immune and reproductive aspects of *Ae. aegypti*. The modified aspects mainly were related to the immune priming enhancement by *Wolbachia* presence and the modulation of the Juvenile Hormone pathway caused by both microorganism’s infection.

## Highlights

● Proteome changes in *Ae. aegypti, Wolbachia, and* ZIKV interactions

● A great diversity of *Wolbachia* proteins were quantified in *Ae. aegypti* ovary

● Mosquito cytoplasmic incompatibility increases during *Wolbachia* and ZIKV infection

● Juvenile Hormone pathway is modulated by both infections

● *Wolbachia* enhances *Ae. aegypti* immune priming mechanism

● ZIKV unsettles the host immune response by reducing antimicrobial peptides production

● Coinfection triggers oxidative stress and a lack of vitellogenin precursors

## 1 Introduction

Zika virus (ZIKV) is a mosquito-borne arbovirus from the family Flaviviridae and is related to other arboviruses such as dengue (DENV), yellow fever (YFV), West Nile (WNV), and Japanese encephalitis (JEV) (Zimler et al., 2021). It was originally reported in a primate from the Zika forest in Uganda in 1947 (Dick et al., 1952). However, the first large outbreak of ZIKV in humans occurred on the Pacific Island of Yap, in the Federated States of Micronesia in 2007 (Cao-Lormeau et al., 2014). In 2014, ZIKV emerged in the Pacific islands and a few years later invaded the Americas, being firstly identified in 2015 in Bahia state, Brazil (Campos et al., 2015). It became the first major infectious disease linked to adverse fetal outcomes, mostly microcephaly in newborns, and other clinical aspects such as acute febrile illness and neurologic complications, for example, Guillain-Barré syndrome. Such outcomes led the World Health Organization to claim a global public health emergency (Cao-Lormeau et al., 2014). Unfortunately, there are no antivirus therapies or vaccines available to mitigate ZIKV transmission, i.e., the development of effective control methods targeting the mosquito vector must be encouraged.

The mosquito *Aedes aegypti* is the main vector of ZIKV worldwide (Chouin-Carneiro et al., 2016; Ferreira-de-Brito et al., 2016). During infection in female mosquitoes, the virus can be located in different tissues, but mainly in the gut, salivary glands, and ovaries (Sá-Guimarães et al., 2021). Ovaries infection is of paramount importance to maintain the vertical transmission of arboviruses, even if at low rates (Thangamani et al., 2016; Manuel et al., 2020; Nag et al., 2021). Therefore, evaluating physiological and molecular changes in mosquito ovaries due to arbovirus infection, especially those affecting life-history traits related to vertical transmission, might provide new insights to design innovative vector control approaches.

Among the strategies to control arbovirus infection in *Ae. aegypti*, the use of the maternally-inherited endosymbiont *Wolbachia pipientis* is proving to be efficient due to a unique combination of phenotypes such as cytoplasmic incompatibility (CI), maternal transmission (MT), and viral-blocking (Moreira et al., 2009; Aliota et al., 2016; Dutra et al., 2016; Ant et al., 2018; Nazni et al., 2019; Indriani et al., 2020; Mancini et al., 2020; Ryan et al., 2020; Gesto et al., 2021; Utarini et al., 2021). Although the bacterium *Wolbachia* is naturally found in >60% of arthropod species, no natural infection is observed in *Ae. aegypti* (Gloria-Soria et al., 2018), which led to experimental transinfection by microinjection of different *Wolbachia* strains into mosquito eggs (McMeniman and O’Neil, 2010). After transinfection, *Wolbachia* is currently located in several tissues and organs of *Ae. aegypti* mosquitoes and the mechanisms resulting in viral blocking are under investigation. Most likely, it involves the activation of the immune system by oxidative stress and downregulation of host proteins involved in pathways that would help to produce resources essential to the virus life cycle (Ford et al., 2020; Martins et al., 2021; Ogunlade et al., 2021; Pimentel et al., 2021).

Besides its effects on arbovirus blocking, many studies showed that *Wolbachia* presence can present multifold effects on *Ae. aegypti* fitness that could later be an additional hurdle for *Wolbachia* establishment. For instance, *Wolbachia*-infected larvae have more rapid development and higher survivorship (Dutra et al., 2016; de Oliveira et al., 2017) but reduced adult size (Ross et al., 2014), delayed embryogenic maturation, and *Wolbachia*-infected *Ae. aegypti* females have lower fecundity, fertility rates, quiescent eggs viability, and vector competence, affecting local invasion patterns (McMeniman and O’Neil, 2010; Dutra et al., 2015; King et al., 2018; Farnesi et al., 2019; Garcia et al., 2019; Allman et al., 2020; Garcia et al., 2020; Lau et al., 2021). Therefore, investigating the interaction network involving mosquito vectors, arboviruses, and *Wolbachia* could help maintain a long-term stable blocking phenotype in endemic regions (Edenbourough et al., 2021).

The proteome can be considered more than a simple translation of the protein-coding regions of a genome, as some post-transcriptional and post-translation modifications generate even millions of proteoforms (Smith et al., 2013). Over the last decades, mass spectrometry-based proteomics has emerged as a powerful tool for identifying and quantifying the proteins contained in a biological sample. It has significantly contributed to unraveling many cellular and organism aspects (Schubert et al., 2017), and highlights features and emergent properties of complex systems under different conditions (Cox and Mann, 2011). To overcome the large overall experimental time, sample consumption, and quantitative variations of proteomics analysis, the isobaric labeling quantitative methods have allowed the examination of multiple samples all at once. Moreover, it increases the throughput of quantification by having a higher multiplex capability, which makes it possible to handle several biological replicates, offering statistical robustness (Chen et al., 2021). A diversity of quantitative methods have already been successfully applied in insects to analyze differentially regulated proteins (De Mandal et al., 2020; García-Robles et al., 2020; Serteyn et al., 2020), including a previous study from our group, where it was able to elucidate different mechanism responses to ZIKV and *Wolbachia* infection in *Ae. aegypti* head and salivary glands (Martins et al., 2021).

This study applies isobaric labeling quantitative mass spectrometry-based proteomics to quantify proteins and identify pathways altered during either ZIKV or *Wolbachia* single infection and coinfection with *Wolbachia/*ZIKV in the *Ae. aegypti* ovaries. We show that it was possible to identify ZIKV peptides and more than 1000 proteins from *Wolbachia* in mosquitoes’ ovaries. The present study offers a rich resource of data that helps elucidate mechanisms by which *Wolbachia* and ZIKV infection in *Ae. aegypti* can interfere in immune and reproductive aspects.

## 2 Material and Methods

### 2.1 Insects

To analyze the *Wolbachia* and ZIKV infection repercussions on *Ae. aegypti* proteome, mosquitoes were used from two different sites in Rio de Janeiro (Rio de Janeiro State, Brazil), with a 13 km distance isolated from each other: Porto (22°53′43″ S, 43°11′03″ W) and Tubiacanga (22˚47’06 “S; 43˚13’32 “W). Porto is an area populated by mosquitoes without *Wolbachia* infection, i.e., wild type. On the other hand, Tubiacanga is the first site in Latin America where a *Wolbachia* (*w*Mel strain) invasion in *Ae. aegypti* was established, with more than 90% frequency (Garcia et al., 2019). Eighty ovitraps were used for egg collection (Codeço et al., 2015) with a net distance of 25-50 m apart. Ovitraps were placed over an extensive geographic area to ensure we captured the local *Ae. aegypti* genetic variability, collecting at least 1500 eggs per site. The eggs were hatched, and the mosquitoes were maintained at the insectary under a relative humidity of 80 ± 5% and a temperature of 25 ± 3°C, with *ad libitum* access to a 10% sucrose solution. Mosquitoes from the F1 generation were selected for experimental infection.

### 2.2 ZIKV Strain and Mosquito Viral Infection

*Aedes aegypti* females were orally infected with the ZIKV strain Asian genotype isolated from a patient in Rio de Janeiro (GenBank accession number KU926309). Local wild *Ae. aegypti* populations have high vector competence to this ZIKV strain (Fernandes et al., 2016; da Silveira et al., 2018; Petersen et al., 2018). The vector competence of the Porto population was assessed in individuals belonging to the same batch as those used herein. We observed that 100% of ZIKV-infected mosquitoes have a disseminated infection on their bodies i.e., the ZIKV infection rate in ovaries was not directly estimated but the whole body (da Silveira et al., 2018). All the assays were performed with samples containing 3.55 × 10^6^ PFU/ml (Martins et al., 2021). The experimental infection followed the protocol described in detail elsewhere (Martins et al., 2021). Briefly, 6-7 days old *Ae. aegypti* females from each of the two populations (Tubiacanga and Porto) were orally infected through a membrane feeding system (Hemotek, Great Harwood, UK), adapted with a pig-gut covering, which gives access to human blood. The infective blood meals consisted of 1 ml of the supernatant of infected cell culture, 2 ml of washed rabbit erythrocytes, and 0.5 mM of adenosine triphosphate (ATP) as phagostimulant. The same procedure and membrane feeding apparatus were used to feed control mosquitoes, but they received a noninfectious blood meal, with 1 ml of cell culture medium replacing the viral supernatant. After the experimental infection, we had a total of 152 *Ae. aegypti* females. Infected mosquitoes were maintained in incubators with a 27 ± 1°C temperature.

### 2.3 Ethical Approval

*Ae. aegypti* colonies with *Wolbachia* are maintained in the lab by blood-feeding of anonymous donors acquired from the Rio de Janeiro State University blood bank. The blood bags were rejected from the bank due to small blood volume. No information on the donors (including sex, age, and clinical condition) was disclosed. The blood was screened for Dengue virus (DENV) using the Dengue NS1 Ag STRIP (Bio-Rad) before use in the mosquito feeding process. The use of human blood was approved by the Fiocruz Ethical Committee (CAAE 53419815.9.0000.5248).

### 2.4 Sample Preparation and LC-MS/MS

A total of 152 *Ae. aegypti* mosquitoes were processed, in which 35 females were *Wolbachia*-infected (W), 42 infected exclusively with ZIKV (Z), 39 were coinfected with both *Wolbachia* and ZIKV (WZ), and 36 were non-infected with those microorganisms (A). At 14 days post-infection (dpi), each mosquito ovary was extracted from the body according to the previous method (Charlwood et al., 2018). Protein extraction, digestion, iTRAQ labeling, and LC-MS/MS protocol were performed as previously described (Martins et al., 2021). Proteins were extracted by lysis with buffer (7 M urea, 2 M thiourea, 50 mM HEPES pH 8, 75 mM NaCl, 1 mM EDTA, 1 mM PMSF) with the addition of a protease/phosphatase inhibitor cocktail (Roche). Lysates were centrifuged, and the supernatants were transferred to new tubes for protein quantification using the Qubit Protein Assay Kit^®^ fluorometric (Invitrogen), following the manufacturer’s instructions. A total of 100 μg of proteins from each condition were processed. Reduction and alkylation steps were performed using 10 mM dithiothreitol (DTT -GE Healthcare) and 40mM iodoacetamide solution (GE Healthcare). Samples were diluted 10x with 50 mM HEPES buffer, reducing the concentration of urea/thiourea, and incubated with trypsin (Promega) in a 1:50 (w/w, enzyme/protein) ratio at 37°C for 18 hours. The resulting peptides were desalted with a C-18 macro spin column (Harvard Apparatus) and then vacuumed dried.

**Figure 1:**
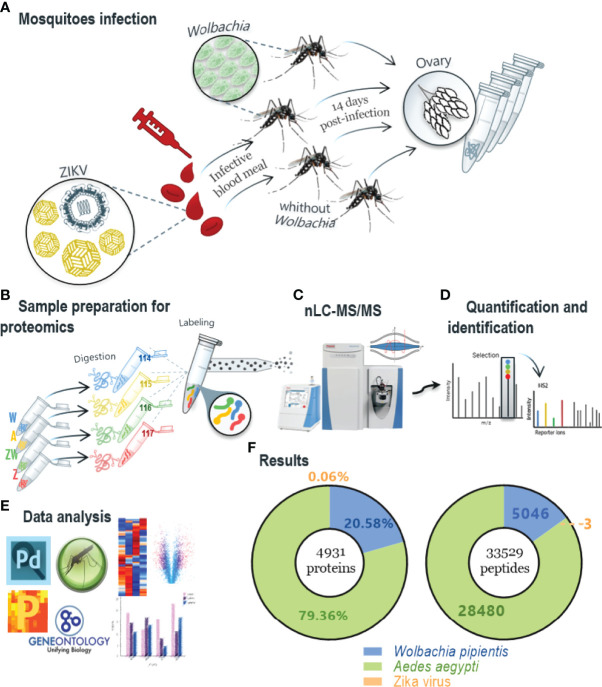
Workflow method and main protein identification results. **(A)** Mosquitoes infection protocol in which mosquitoes infected and non-infected with Wolbachia were captured and half were infected with ZIKV in vitro; ovaries were extracted after 14 days. **(B)** Sample preparation for proteomics, using trypsin enzyme for digestion step and peptides labeling was performed with iTRAQ-4plex and mixed in a 1:1:1:1 ratio; labeled peptides were fractionated offline using HILIC chromatography; samples were named depending on its infection condition: A - non-infected mosquitoes; W - Wolbachia infected; Z - ZIKV infected. **(C, D)** The pool of fractions was analyzed by nLC–MS/MS in Q-Exactive Plus mass spectrometer and the fragmentation scheme shows reporter ions at the low m/z region used for relative quantification of the peptides/proteins. **(E)** Data analysis was first performed with Proteome Discoverer 2.4 software using Ae. aegypti, Wolbachia and ZIKV peptides database and after statistical and bioinformatics analysis was done in software Perseus using VectorBase to enrich proteins differentially regulated for GO and KEGG. **(F)** Summary proteins identification result, where a total of 4931 proteins and 33,529 peptides were identified, including the three organisms.

Peptides were labeled with isobaric tags for relative and absolute quantitation (iTRAQ) 4-plex (ABSciex), performed as described in Martins et al. (2021), mixing the tag solutions with sample peptides at room temperature for one hour. Each condition was labeled as follows i) Tag 114 corresponded to sample W (*Wolbachia* infected); ii) Tag 115 to sample A (none infection); iii) Tag 116 to sample WZ (*Wolbachia* and ZIKV coinfection); and iv) Tag 117 to sample Z (ZIKV infection). After the labeling, samples were combined and vacuum dried for offline fractionation by HILIC (hydrophilic interaction liquid chromatography) prior to LC-MS/MS analysis. The dried samples were resuspended in acetonitrile (ACN) 90%/trifluoroacetic acid (TFA) 0.1% and injected into Shimadzu UFLC chromatography using a TSKGel Amide-80 column (15 cm x 2 mm i.d. x 3 μm - Supelco), which was equilibrated using ACN 85%/TFA 0.1% (phase A). The peptides were eluted in TFA 0.1% (phase B), and every eight fractions were collected and combined according to the separation and intensity of the peaks. The pools of fractions were dried in a speed vac and resuspended in 0.1% formic acid (FA). LC-MS/MS analysis was performed in an Easy-nLC 1000 coupled to a Q-Exactive Plus mass spectrometer (Thermo Scientific, Waltham, MA, USA). Ionization was performed in an electrospray source with the acquisition of spectra in positive polarity by data-dependent acquisition (DDA) mode, spray voltage of 2.5 kV, and temperature of 200°C in the heated capillary. The acquisition was set as follows: full scan or MS1 in the range of 375 - 1800 m/z, resolution of 70,000 (m/z 200), fragmentation of the 10 most intense ions in the HCD collision cell, with standardized collision energy (NCE) of 30, resolution of 17,000 in the acquisition of MS/MS spectra, the first mass of 110 m/z, isolation window of 2.0 m/z and dynamic exclusion of 45 s. A description of the workflow is presented in **Figure 1**.

### 2.5 Data Analysis and Gene Enrichment

The spectra obtained after the LC-MS/MS analyses were processed in the Proteome Discoverer 2.4 Software (Thermo Scientific), with the Sequest HT search engine against the *Ae. aegypti* (genome version/assembly ID: INSDC: GCA_002204515.1), ZIKV and *Wolbachia* provided by VectorBase (Giraldo-Calderón et al., 2014), ViPR (Pickett et al., 2012), and UniProt (The UniProt Consortium, 2015). 2019), respectively. For the search, the following parameters were used: precursor tolerance of 10 ppm, fragment tolerance of 0.1 Da, tryptic cleavage specificity, two maximum missed cleavage sites allowed, fixed modification of carbamidomethyl (Cys), variable modification of iTRAQ 4-plex (Tyr, Lys, and peptide N-terminus), phosphate, (Ser, Thr, and Tyr) and oxidation (Met). Peptides with high confidence were selected, and only identifications with q values equal to or less than 0.01 FDR were considered. Target Decoy PSM obtained these values.

Quantitative analysis was performed in Perseus software, version 1.6.12.0 (Tyanova et al., 2016), based on the intensity of the reporter ions extracted from the MS/MS spectra. All data were transformed into log2 and normalized by subtracting the column median. As a criterion to define the differential proteins, statistical Anova tests were performed between the groups, with a significance level of 0.05 of the p-value. Proteins with p < 0.05 were considered significant with a fold change threshold of 0.5. Enrichment of biological process by gene ontology and metabolic pathways by KEGG for differentially expressed proteins was performed using Fisher’s Exact Test (p<0.05) on the VectorBase website (bit.ly/38OmEX0) (Ashburner et al., 2000; Giraldo-Calderón et al., 2014).

## 3. Results and Discussion

### 3.1 Proteome Identification and Quantification

Isobaric-labeled quantitative proteomics was applied to increase proteomics analysis’s depth coverage and efficiency. By labeling mosquitoes from each of the four experimental groups with iTRAQ 4-plex, samples were multiplexed before offline fractionation and LC-MS/MS analysis (**Figure 1**). We identified 4931 protein groups (**Supplementary Table S2**), 36,403 peptides, 110,733 PSMs, and 987,698 MS/MS spectra. The protein search was performed using *Ae. aegypti*, ZIKV, and *Wolbachia* databases, simultaneously. Considering all identified proteins, 3913 belong to *Ae. aegypti*, 1015 to *Wolbachia*, and three unique peptides with a total of three PSMs to the ZIKV polyprotein.

We contrasted our proteomic dataset of *A. aegypti* ovary with the ovary proteome obtained by Geiser et al., 2022. For the analysis, we initially performed conversion of the identifiers because the database used by the authors is from a different origin and version (**Supplementary Table S1**). We found that the database used comprised more than one genome deposited in VectorBase. Therefore, we compared the gene IDs also by orthology and identified 1291 genes in common between both works (**Supplementary Figure S1**). It is essential to highlight that Winzerling’s paper used a distinct sample and MS analysis method compared to this work.

**Figure 2:**
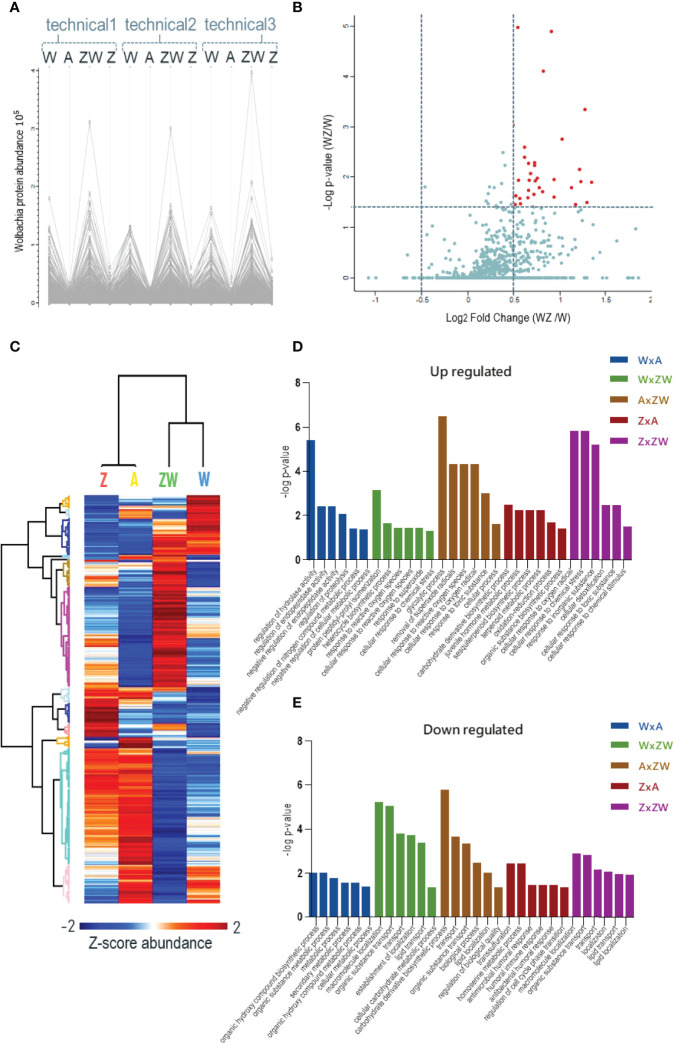
Quantitative proteomics data. **(A)** Abundance chart of the three replicates of identified *Wolbachia pipientis* proteins. High expression is observed in the W and ZW samples, showing that it is possible to compare the expression of proteins by the relative quantification of the iTRAQ; **(B)** Volcano plot of *Wolbachia* proteins comparing WZ/W. The red dots represent upregulated proteins; **(C)** Heatmap of all Ae aegypti identified proteins that were upregulated (red) or downregulated (blue) during the comparison between control, monoinfected, and coinfected samples; **(D)** Mainly upregulated pathways statistically relevant in each condition comparison. **(E)** Mainly downregulated pathways statistically relevant in each condition comparison.

After checking the quantification using the report ion signals’ abundance (**Figure 2A**), it is possible to observe that in samples A and Z, the signals’ abundance is very low (due mostly to isotopic contamination in tags) and shows the intensities that account for overlapping isotopic contributions of the reagent purity values, showing the signal-to-noise (S/N) behavior of iTRAQ reporter ions for the channels where *Wolbachia* protein signals are not expected. This control is critical to show that *Wolbachia* protein identifications were not random matches. Instead, they followed the expected quantification trend: only relevant signals within *Wolbachia* infection channels. Since in W and WZ, where we have *Wolbachia* infected samples, there are higher intensity and relevant signals of bacterium proteins revealing the match between identification and quantification pattern of *Wolbachia* proteins.

#### 3.1.1 Wolbachia Localization in Insect Ovary and Its Protein Identification

The accumulation of *Wolbachia* in ovaries improves its vertical transmission efficiency through the female germline suggesting that a balance between vertical transmission and optimum densities is critical for a long-term, stable endosymbiosis (Kaur et al., 2021). Since only 1205 protein-coding genes were reported in the *Wolbachia pipientis* genome (Sinha et al., 2019), we applied an identification strategy using a combined database containing Uniprot proteins addressed to *Wolbachia* species in general. Using this strategy, we were able to identify 1015 unique protein groups from *Wolbachia*. As an additional analysis, we compared W and WZ to observe possible *Wolbachia* proteins that might be modulated during ZIKV infection (**Figure 2B**). Proteins with p < 0.05 were considered significant with a fold change threshold of 0.5. A total of 38 proteins were upregulated in response to virus infection (**Table 1**), mostly related to catalytic activity, structure-activity, metal ion binding, nucleotide binding, and RNA and DNA binding. Similar findings were noted after evaluating that the transcriptional response of *Wolbachia* to DENV involves the regulation of *Wolbachia* DNA production and energetic metabolic genes (Leitner et al., 2021).

**Table 1:** Wolbachia proteins with significant differences in abundance between W and WZ.

Uniprot	Proteins	p-value	Fold changes W/WZ	Biological Process	Cellular Component	Molecular Function
Q73IW9	Bifunctional DNA-directed RNA polymerasesubunitbeta-beta'	0,000444461	0,411035542	metabolic process		catalytic activity;DNA binding
Q73H30	Porin 4 domain-containing protein	0,012058539	0,595912427			
A0A5R9MDU9	ATP-dependentchaperoneClpB	0,002478004				
A0A5R9MBZ5	Insulinase family protein	0,04901528	0,579255197			
Q73G77	Outer membrane protein assembly factor BamA	0,018472215	0,527555786	cell organization and biogenesis	membrane	
Q73HD5	Cytoplasmic incompatibility factor CifA	779842E-05	0,560087056			
Q73H52	Hypothetical protein	0,024988487	0,51835011		membrane	
AQA098ASI2	Pyruvate, phosphatedikinase	1,05157E-05	0,579445156	metabolic process		catalyticactivity;metal ion binding;nucleotide binding
A0AO98ASBS	Ribosome-recycling factor	0,021748863	0,503098093	cell organization and biogenesis;metabolicprocess	cytoplasm	RNA binding
Q73HB3	TranscriptionelongationfactorGreA	0,034470463	0,440927891	metabolic process;regulation of biological process		DNAbinding;proteinbinding;RNAbinding
Q4ECB1	OmpA family protein	0,001739336	0,485774855		membrane	
Q73HQ7	Surface antigen, Wspparalog	0,011469895	0,62249352			
A0A09SAS15	MgtEintracelularN domain	0,000908949	0,704846358			
M9WUJ8	SOS ribosomal protein L3	0,026263219	0,67093336	metabolic process	ribosome	RNAbinding;structural molecule activity
C0F8V0	Phosphatidylserine decarboxylase proenzyme	0,045093524	0,427720959	metabolic process	membrane	catalytic activity
Q4EAL9	50SribosomalproteinL1	0,034407904	0,666474541	metabolic process;regulation of biological process	ribosome	RNAbinding;structural molecule activity
Q73HX6	Aspartate-tRNA(Asp/Asn) ligase	0,005344562	0,52587834	metabolic process	cytoplasm	catalytic activity;nucleotide binding
Q73FS0	ATP-dependent zinc metalloprotease FtsH	0,010445494	0,585365863	cell division; metabolic process;response to stimulus	membrane	catalyticactivity;rnetal ion binding;nucleotide binding
A0A5B9K3B3	SOS ribosomal protein L5	0,039504806	0,505903393			
A0AQ98ATK8	Citrate synthase	0,011127846	0,515264518	metabolic process	cytoplasm	catalytic activity
C0F9Z9	Protein HfIC	0,031868241	0,403411331	regulation of biological process	membrane	
A0A2A4IJ89	Inositol monophosphatase	0,005206978	0,597804797			
Q73HW7	Isoleucine-tRNA ligase	0,00713402	0,427841423	metabolic process;regulation of biological process	cytoplasm	catalytic activity;metal ion binding;nucleotidebinding;RNA binding
AOA060Q1B9	Iron(lll) transportsystem substrate-binding protein	0,023415784	0,589204769	transport		metal ion blnding;transporter activity
Q73GT7	Hypothetical protein	0,01601564	0,452984557			
A0A1V2N3U3	Leucine-tRNA ligase	1.26546E-05	0,529255411	metabolic process;regulation of biological process	cytoplasm	catalytic activity;nucleotide binding
Q73GS5	Type IV secretion system protein VirB6	0,00857789	0,61741491	transport	membrane	
A0A2A4IGS0	ATPsynthaseganmachain	0,042213912	0,574342268			
A0AQ98ARZ7	Fido domain-containing protein	0,011555349				
A0A5R9MDS2	Folate-bindingproteinYgfZ	0,042029907	0,7052091			
C0F8P8	MalonylCoA-acylcarrierproteintransacylase	0,005819131	0,598572843	metabolic process		catalytic activity
A0A225X8G2	DNA mismatch repair protein MutS	0,012269948	0,422355559			
Q4ECU7	NADH-quinoneoxidoreductasesubunitC	0,025713881	0,631272563	metabolic process;transport	membrane	catalytic activity
M9WVR3	ReplicativeDNA helicase	0,015991817	0,575902317	metabolic process		catalyticactivity;DNAbinding;nucleotide binding
A0A2A4IK13	Magnesium transporter MgtE	0,049013747	0,435309293			
B7TW57	RmlD_sub_bind domain-containing protein (Fragment)	0,034994832	0,592799831			
I7IU09	Putative pyruvate, phosphate dikinase regulator/ protein	0,004012121	0,545064889	metabolic process		catalytic activity;nucleotide binding
B5Y8B7	Ankyrinrepeatdomainprotein	0,012561568	0,38957124			nucleotide binding;protein binding
A0AO98AU34	Peptidyl-prolyl cis-trans isomerase	0,019230457	0,562841529	metabolic process	membrane	catalytic activity

One of the positively modulated proteins in WZ compared to W was the Cytoplasmic incompatibility factor (CifA). LePage et al. (2017) concluded that the cytoplasmic incompatibility factor genes enhanced the cytoplasmic incompatibility leading to embryonic lethality in *D. melanogaster*. The gene additively, through transgenic mechanisms, augments embryonic lethality in crosses between infected males and uninfected females after pioneering genetic studies. The discovery of cifA pioneers genetic studies of prophage WO-induced reproductive manipulations and informs the continuing use of *Wolbachia* to control dengue and Zika virus transmission to humans.

#### 3.1.2 ZIKV Polyprotein Peptides Were Identified in Mosquito Ovaries

A total of three ZIKV peptides were identified in mosquito ovaries using the Isobaric-labeled quantitative proteomics approach: VPAETLHGTVTVEVQYAGNDGPCKIPVQMAVDMQTLTPVGR, NGGYVSAITQGRREEETPVECFEPSMLK, and DGDIGAVALDYPAGTSGSPILDR (**Supplementary Figure S2**). However, no protein quantification data were obtained despite its borderline identification in ovary tissue. This result leads to two different hypotheses: ZIKV was not located in mosquito ovaries, or a low amount of ZIKV polyprotein peptides was detected in LC-MS/MS analysis leading to a poor quantification in this tissue by the method used.

Vector competence is a crucial feature in determining the likelihood of pathogen transmission in a given area and has hitherto shown heterogeneous results among field populations (Epelboin et al., 2017; Kauffmann and Kramer, 2017; Boyer et al., 2018). ZIKV disseminates through several organs and tissues of mosquitoes, including the ovaries, to support further vertical transmission (Campos et al., 2017; Li et al., 2017; González et al., 2019; Lai et al., 2020; Sá-Guimarães et al., 2021). The infection of ovaries by ZIKV is dependent primarily on mosquito genetics, temperature, viral load, and the availability of blood meals but peaks around 18 dpi (Thangamani et al., 2016; Nag et al., 2021). Sá-Guimarães et al. (2021) identified ZIKV in *Ae. aegypti* ovaries at 3 dpi after orally challenging insects with a 2.5×10^9^ PFU/ml viral load, whereas our ZIKV isolate was 3.55×10^6^ PFU/ml. Intuitively, the higher viral load used by Sá-Guimarães et al. (2021) and the long incubation period before ovaries dissection by Nag et al. (2021) together might explain the lack of ZIKV peptides quantification in *Ae. aegypti* ovaries in our study. Nevertheless, we achieved ZIKV identification in ovaries and revealed that ZIKV mono-infection modulated several proteins in *Ae. aegypti* ovaries that will be further discussed.

### 3.2 Modulated Proteins and Pathways by Infections

A total of 480 proteins from *Ae. aegypti* with significant differences were statistically determined by the ANOVA test. Using the Tukey post-test ANOVA, we defined pairs of proteins with significant differences between the groups (**Supplementary Table S2**) (**Figure 2C**). Pathways were enriched using VectorBase software and the Gene Ontology/KEGG list is represented in **Supplementary Table S3**. Pathways related to reproductive and immune aspects were the main interest in this data and were the focus of discussion (**Figures 2D, E**).

#### 3.2.1 ZIKV Mono-Infection Controls Mosquito Machinery to Favor Its Replication and Transmission

##### 3.2.1.1 ZIKV Infection Downregulates Antimicrobial Peptides and Mannose-Binding C-Type Lectin

During the investigation of modulated proteins in ZIKV mono-infection samples compared to control, it was possible to identify attacin (AAEL003389), included in the antimicrobial humoral response (GO:0019730), humoral immune response (GO:0006959), and antibacterial humoral response (GO:0019731) pathways, and gambicin (AAEL004522) downregulation. Both molecules are classified as antimicrobial peptides (AMPs), crucial effectors of the insect’s innate immune system that can provide the first line of defense against various pathogens (Wu et al., 2018). It is widely known that AMPs are normally produced by the Toll/IMD signaling pathway, which is the case of attacins (Buonocore et al., 2021), or can also be produced by JAK-STAT pathway, as gambicin (Zhang et al., 2017). The probable reason for its downregulation is the influence of AMPs in virus infection and the difficult DENV and ZIKV establishment in *Ae aegypti* (Xi et al., 2008; Martins et al., 2021). In addition to attacin and gambicin, RpS23: 40S ribosomal protein S23 (AAEL012686) was also downregulated, and it was recently discovered that new AMP functioning as they are capable of recognizing microorganisms molecules, and an effector, capable of killing the potential pathogens (Ma et al., 2020).

Besides AMPs, a mannose-binding C-type lectin (AAEL000563) was also downregulated. C-type lectins (CTLs) are a family of proteins that contain characteristic modules of carbohydrate recognition domains and play important roles in insect immune responses, such as opsonization, nodule formation, agglutination, encapsulation, melanization, and prophenoloxidase activation (Xia et al., 2018). A more specific function related to mannose-binding lectins is a pattern recognition component of the complement system that binds carbohydrate groups on the surface of microbial pathogens triggering the lectin activation pathway of complement (Takahashi et al., 2006). It is described that this pathway can neutralize DENV and West Nile Virus (WNV) infection (Avirutnan et al., 2011; Fuchs et al., 2011). Moreover, it was recently described that CTLs can act as a recognition receptor for JAK-STAT immune pathway (Geng et al., 2021), which downregulation may interfere in AMPs production, as gambicin peptide. Those downregulations combined can facilitate ZIKV infection.

##### 3.2.1.2 ZIKV Enhance Juvenile Hormone Production and Pro-Viral Host Factors for Establishing Infection

Juvenile Hormone (JH), a representant from a family of sesquiterpenoid hormones in insects, was originally described in *Rhodnius prolixus* as a molecule capable of maintaining the juvenile character of insect larvae to ensure proper metamorphosis timing (Wigglesworth, 1934). However, JHs govern many insects’ essential aspects of development, metamorphosis, and reproduction (Tsang et al., 2020). Its absence in vertebrates may qualify this hormone as a target to control insect pests and disease vectors (Jindra et al., 2015). Its signaling and production pathways comprehend the interaction between insects’ neuronal and fat body organs, and the main effect in female ovaries is the vitellogenin production activation (Santos et al., 2019). Besides, Chang et al. (2021) recently described that JH acts in AMP negative regulation, especially after the post-eclosion phase of the *Ae. aegypti* female gonadotrophic reproductive cycle. In regard to *Ae. aegypti*, it was described that JH analogs enhance ZIKV infection (Alomar et al., 2021). On the other hand, the silencing of ribosomal protein (Rp) genes, responsive to JH, increased ZIKV blocking once the virus increased global ribosomal activity in the insect (Shi et al, 2021). Taking all of that information into account, it is vital to understand JH modulation during ZIKV infection and if *Wolbachia* infection plays any changes as well.

**Figure 3:**
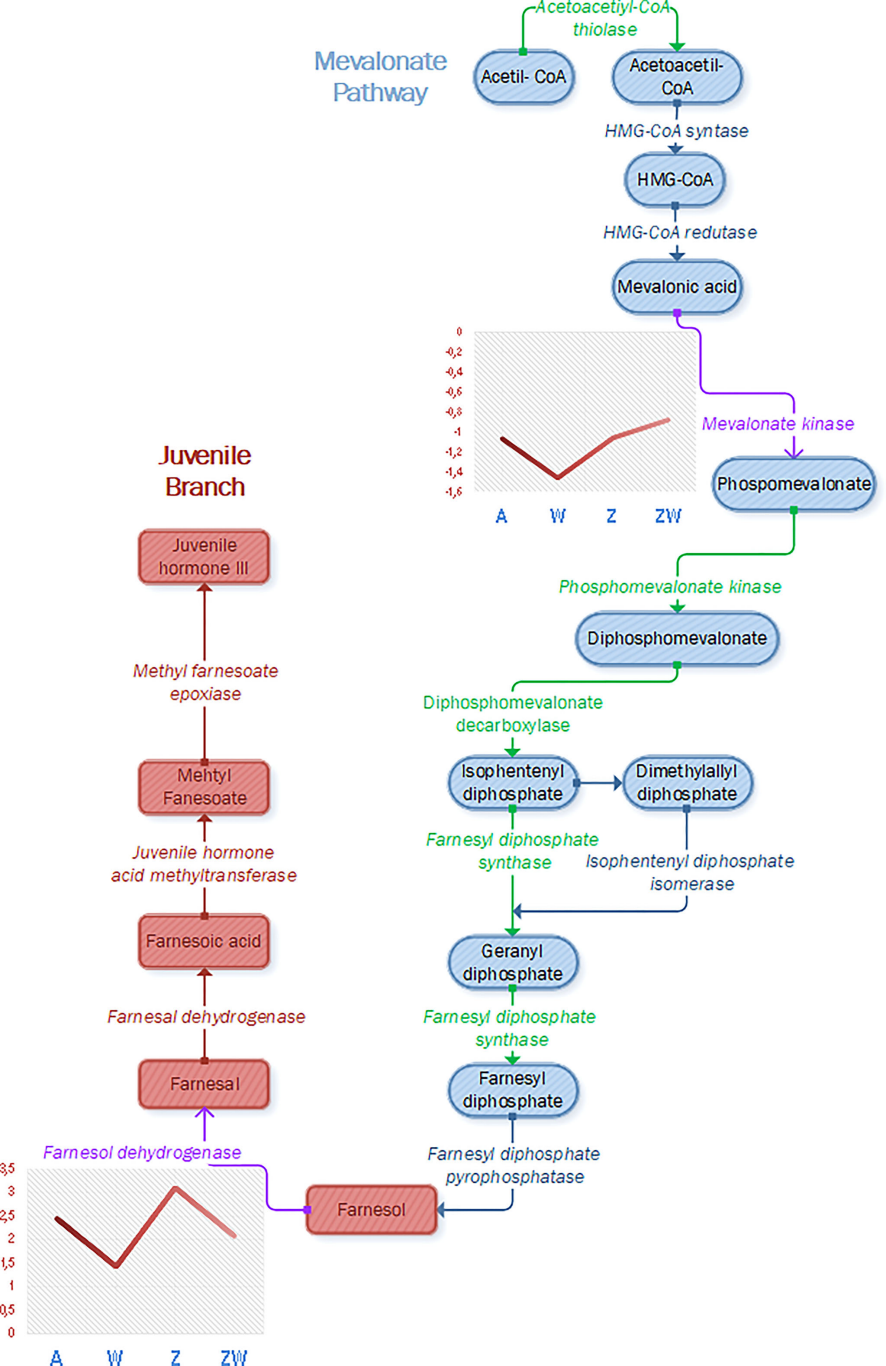
Identification and modulation of proteins that participate in the JH pathway, composed of the mevalonate pathway (early step), represented in gray, and juvenile hormone branch (last step), represented in blue, and juvenile hormone branch (last step), represented in red (pathway adapted from Nouzova et al., 2011). Enzymes colored in green were identified in *Ae. aegypti* proteome in this work, and enzymes colored in purple were identified and quantified as modulated during ZIKV or *Wolbachia* infection. Mevalonate kinase and farnesol dehydrogenase were downregulated in *Wolbachia* infection and farnesol dehydrogenase was upregulated in ZIKV infection, as represented in graphs containing each condition and the respective fold change of each modulated protein.

Evaluating our data, the first aspect that will be discussed is ZIKV upregulation in the JH production pathway, as a farnesol dehydrogenase (AAEL017302) was upregulated (**Figure 3**). Farnesol dehydrogenase was found in *Ae. Aegypti* (Mayoral et al., 2009), and its activity is observed on the second JH branch, oxidizing farnesol to farnesal (Nouzova et al., 2011). It is considered a rate-limiting enzyme and critical in regulating the production of JH in adult mosquitoes (Zifruddin et al., 2021), so its enhancement in ZIKV infection can lead to JH production, as previously described in the literature and exposed in the last paragraph, favoring mosquito reproduction aspects and maybe helping ZIKV vertical and horizontal transmission. Experiments involving farnesol dehydrogenase inhibition showed larvicidal activity and inhibited the ovary growth of female *Ae. albopictus* (Park et al., 2020). Farnesol dehydrogenase modulated several pathways in our analysis: juvenile hormone metabolic process (GO:0006716), cellular hormone metabolic process (GO:0034754), juvenile hormone biosynthetic process (GO:0006718), sesquiterpenoid biosynthetic process (GO:0016106), terpenoid biosynthetic process (GO:0016114), terpenoid metabolic process (GO:0006721), sesquiterpenoid metabolic process (GO:0006714), hormone metabolic process (GO:0042445), hormone biosynthetic process (GO:0042446), regulation of hormone levels (GO:0010817), oxidation-reduction process (GO:0055114), cellular biosynthetic process (GO:0044249), and organic substance biosynthetic process (GO:1901576).

ZIKV-capsid interaction with cell host was newly investigated through quantitative label-free proteomics and showed an important contribution to the 26S protease regulatory subunit (Gestuveo et al., 2021), a component of the ubiquitin-proteasome system. We were able to identify two 26S protease regulatory subunit (AAEL002508 and AAEL012943) upregulated, related to protein catabolic process (GO:0030163), regulation of proteolysis (GO:0030162), positive regulation of catabolic process (GO:0009896), positive regulation of cellular protein catabolic process (GO:1903364), positive regulation of proteolysis involved in cellular protein catabolic process (GO:1903052), positive regulation of protein catabolic process (GO:0045732), positive regulation of proteasomal protein catabolic process (GO:1901800), positive regulation of cellular catabolic process (GO:0031331), regulation of proteolysis involved in cellular protein catabolic process (GO:1903050), regulation of cellular protein catabolic process (GO:1903362), regulation of proteasomal protein catabolic process (GO:0061136), positive regulation of proteolysis (GO:0045862), regulation of protein catabolic process (GO:0042176), and macromolecule catabolic process (GO:0009057). It is already described 26S protease regulatory subunit importance in ZIKV infection but the mechanism is currently unknown, but there is a strong relation with ubiquitination processes in other viruses and can help in viral capsid transport to the nucleus, for example (Schneider et al., 2021). Moreover, this ribosomal activity increases the proteolytic activity of the proteasome, which is required for female insect reproduction (Wang et al., 2021), it is sensible to JH and enhances viral replication in *Ae. aegypti* as observed by Shi et al. (2021). Two ribosomal proteins, RpL27a (AAEL013272) and RpL17 (AAEL000180), were also upregulated, related to the organonitrogen compound biosynthetic process (GO:1901566), cellular biosynthetic process (GO:0044249), and organic substance biosynthetic process (GO:1901576).

#### 3.2.2 *Wolbachia* Harms Mosquito Reproductive Characteristics But Helps Its Immune System

##### 3.2.2.1 Juvenile Hormone Pathway Is Negatively Affected by *Wolbachia* Mono-Infection

**Figure 4:**
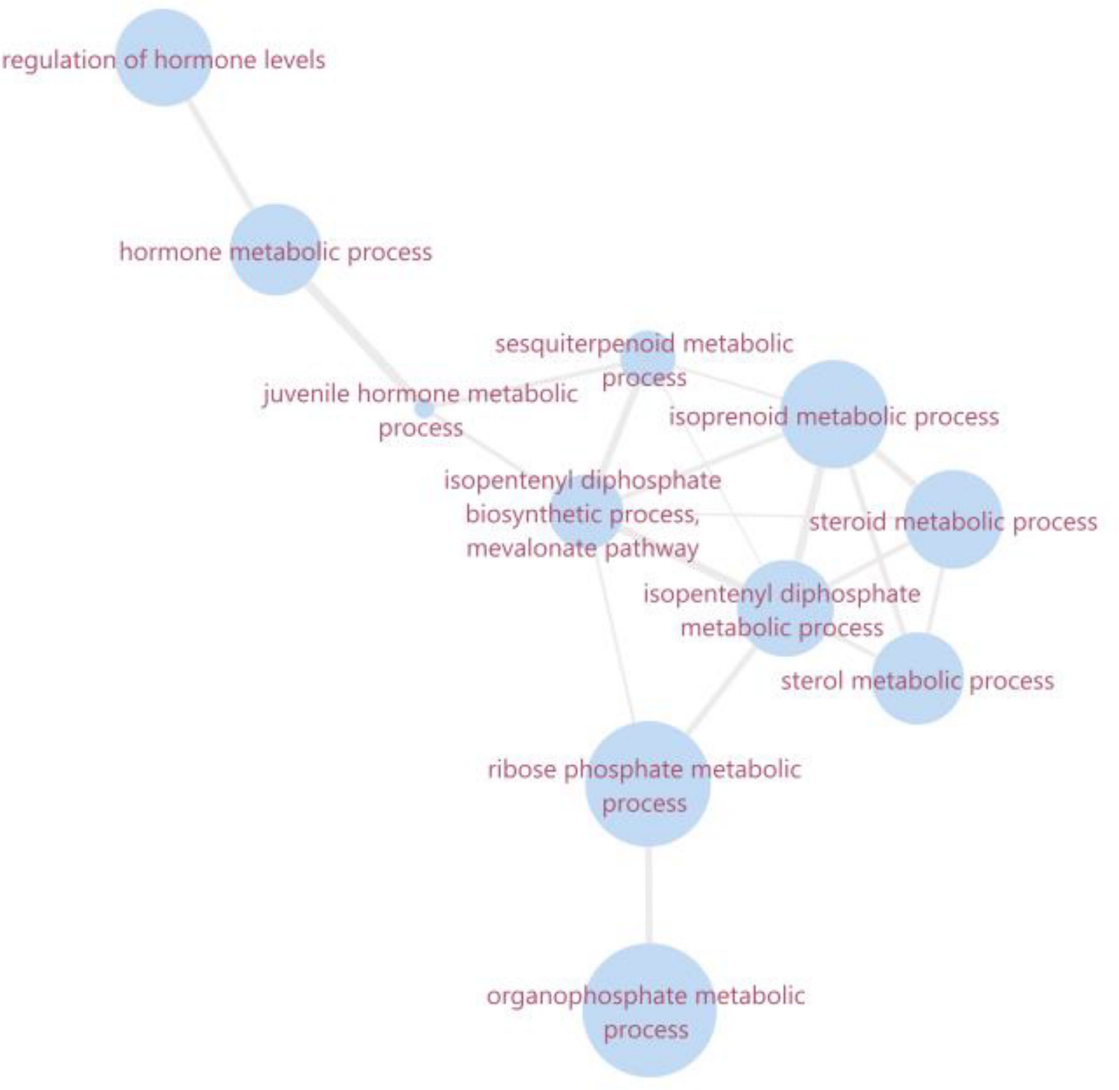
Ontology network of summarized enriched biological processes related to mevalonate kinase and farnesol dehydrogenase. The size of the bubble corresponds to the LogSize value for the GO Term. The overview and the interaction network were obtained in software revigo and cytoscape 3.8.0, respectively.

While ZIKV infection induces JH, *Wolbachia* infection downregulates two proteins related to its pathway (**Figure 3**). One of them is in the same farnesol dehydrogenase (AAEL017302) discussed before, which will affect the second part of JH output cascade. The other protein identified as downregulated is mevalonate kinase (AAEL006435). Mevalonate kinase belongs to the fourth reaction step of the mevalonate pathway, which is responsible for the biosynthesis of many essential molecules important in insect development, reproduction, chemical communication, and defense (Li et al., 2016). Concerning JH, the mevalonate pathway is classified as the early step that forms farnesyl pyrophosphate used on the late step, known as JH branch (Noriega, 2014). Both mevalonate kinase and farnesol dehydrogenase downregulation in *Wolbachia* presence may infer that JH production is negatively affected. This can impact the germline lifecycle from meiosis to gametogenesis, once it was found that JHs influence embryonic reproductive development (Barton et al., 2021). Both farnesol dehydrogenase and mevalonate kinase have modulated pathways that interact with themselves and are related to hormone synthesis (**Figure 4**).

Allied to this event, a yellow protein was also downregulated. Yellow protein (dopachrome conversion enzyme) is involved in the melanin biosynthetic pathway that significantly accelerates pigmentation reactions in insects and belongs to a rapidly evolving gene family generating functionally diverse paralogs with physiological functions still not understood (Noh et al., 2015). Noh et al. (2020) discovered that two yellow proteins are required for egg desiccation resistance, so maybe *Wolbachia* downregulation may decrease egg resistance leading to a lower reproduction success, which was described before (McMeniman et al., 2011).

##### 3.2.2.2 Host Immune Defense Is Activated by *Wolbachia* Mono-Infection as a Protection Barrier to Other Microorganisms

Microorganisms are important modulators of host phenotype, providing heritable variation upon which natural selection acts (Brinker et al., 2019). Host-parasite interactions represent one of the strongest selection pressures in nature, with a considerable impact on the ecology and evolution of parasites and thus on disease epidemiology (Bose and Schulte, 2014). The endosymbiotic bacteria *Wolbachia* has shown an increase in host protection range against pathogens, including bacteria, viruses, nematodes, and the malaria parasite (Wong et al., 2011). A single mechanism called immune priming might explain this broad-based pathogen protection. *Wolbachia* presence upregulates the basal immune response, preparing the insect to defend against subsequent pathogen infection (Ye et al., 2013).

Our results showed that macroglobulin complement protein (AAEL001794) was upregulated during *Wolbachia* infection compared to control. Macroglobulin complement-related factor (MCR) is known for being a thioester-containing proteins (TEP) family component in insects, presenting a protease inhibitor activity (Blandin, 2004). TEPs were previously described in *Anopheles gambiae* and *Drosophila melanogaster* models, and act in the innate immune response by promoting recruitment of immune cells, phagocytosis, and direct lysis of microbial invaders (Shokal & Eleftherianos, 2017), usually in insects hemolymph (Cheng et al., 2016; Mukherjee et al., 2019). Xiao et al. (2014) performed molecular biology assays evolving MCR in *Ae. aegypti* containing *Wolbachia* during DENV infection, describing that MCR does not directly interact with the flavivirus, requiring a mosquito homolog of scavenger receptor-C (SR-C), which interacts with DENV and MCR simultaneously (*in vitro* and *in vivo*) and this SR-C/MCR axis regulates the expression of antimicrobial peptides (AMPs) consequently increasing anti-DENV immune response.

In addition to MCR, three leucine-rich repeat immune proteins (LRRIM) were upregulated during *Wolbachia* infection: LRRIM1, LRRIM8, and LRRIM10A (AAEL012086, AAEL001420, AAEL001401, respectively). LRRIM is evolutionarily conserved among many proteins correlated with innate immunity in an array of organisms, including invertebrates, and it usually forms a disulfide-bridged complex that interacts with the third factor, TEP, more specifically TEP1, revealing its antimicrobial activity in insect immune response (Waterhouse et al., 2010). More importantly, it was noticed that LRRIM responded to ZIKV infection in *Ae. aegypti* female adults in a transcriptional analysis approach (Zhao et al., 2019; Shi et al., 2021), therefore corroborating the idea that LRRIM enhancement mediated by *Wolbachia* presence can help in ZIKV block.

A DEAD box ATP-dependent RNA helicase (AAEL008738) was also upregulated during bacterial infection. DEAD-box helicases are a large family of conserved RNA-binding proteins that belong to the broader group of cellular DExD/H helicases, with emerging evidence that it plays a role in the recognition of foreign nucleic acids and the modulation of viral infection (Taschuk & Cherry, 2020). DEAD-box helicases can play an essential role in sensing viral infection and directly affecting virus RNA by participating in the RNAi pathway and Toll-like and retinoic acid-inducible gene-I-like receptors signaling pathway, for example (Ahmad & Hur, 2015; Baldaccini & Pfeffer, 2021; Su et al., 2022). However, there is evidence that noncoding subgenomic flavivirus RNA from ZIKV can bind to DEAD/H-box helicase ME31B in *Ae. aegypti* as a way of overcoming this defense (Göertz et al., 2019).

### 3.4 Microorganisms Coinfection Generate Oxidative Stress and Affect Yolk Component

#### 3.4.1 Immune Response Mediated by Reactive Oxygen Species and DExD/H Helicases

Martins et al. (2021) earlier described that *Wolbachia* and ZIKV coinfection increased host cellular aerobic metabolism to accumulate reactive oxygen species (ROS) in *Ae. aegypti* head and salivary glands quantitative proteomics. Several pathways related to aerobic metabolism were upregulated in mosquito ovaries analysis, thus corroborating previous data. The increase in aerobic respiration metabolism has already stimulated ROS production in insect cells as part of the host immune response, but it is counterbalanced by the activation of antioxidant pathways (Zug & Hammerstein, 2015; Martins et al., 2021). ROS accumulation activates the mosquito Toll immune pathway, resulting in the production of AMPs (Pan et al., 2012). Possibly as a result of this ROS amount, Cu-Zn superoxide dismutase (SOD) proteins (AAEL019937 and AAEL025388) were upregulated comparing WZ with W, Z, and A. SODs are important antioxidant enzymes that convert superoxide into oxygen and hydrogen peroxide (Park et al., 2012; Lomate et al., 2015) and it is also related to protecting insects’ ovaries during diapauses (Sim and Denlinger, 2011). Besides ROS-mediated immune response, a DEAD box ATP-dependent RNA helicase (AAEL004978) was upregulated in coinfection. DExD box RNA helicase was already discussed in *Wolbachia* mono-infection upregulations and leads us to believe that it is a *Wolbachia*-mediated attempt to overcome ZIKV infection, even though this virus has a way of overcoming this immune mechanism (Göertz et al., 2019).

#### 3.4.2 Vitellogenin A1 Downregulation During Coinfection

During WZ comparison with Z, W, and A, it was noticed that vitellogenin A1 (AAEL010434) was downregulated in all analyses, related to macromolecule localization (GO:0033036), organic substance transport (GO:0071702), biological process (GO:0008150), transport (GO:0006810), the establishment of localization (GO:0051234), localization (GO:0051179), lipid transport (GO:0006869), and lipid localization (GO:0010876). Vitellogenins are the predominant yolk protein precursors produced extra ovarian and taken up by the growing oocytes against a concentration gradient (Engelmann, 1979). By having this important role, it is relevant to understand how it behaves during microorganisms’ infection. Depending on which *Wolbachia* is allocated in the host, the ovary protein level can change, resulting in different protein content in embryos (Christensen et al., 2016), nonetheless, there is a report that establishes a link between the vitellogenin-related mode of transovarial transmission and efficient maternal transmission of *Wolbachia*, assuming that the bacteria utilized vitellogenin transportation system to enter the insect ovaries (Guo et al., 2018). Vitellogenesis can be directly regulated by insect hormones, including JH (Shapiro and Taylor, 1982; Wu et al., 2021). As was discussed before, *Wolbachia* decreases enzyme levels of the JH pathway, and it might interfere in vitellogenesis, together with the yellow protein downregulation.

On the other hand, female mosquitoes with regular blood-feeding presented high oviposition, probably mediated by nutrition influence in hormones, and it enhanced ZIKV infection (Rocha-Santos et al., 2021). This may lead to comprehension of reproduction–immunity trade-offs in insects. Immune defense and reproduction are physiologically and energetically demanding processes. They have been observed to trade off in a diversity of female insects: increased reproductive effort results in reduced immunity, and reciprocally, infection and activation of the immune system reduce reproductive output (Schwenke et al., 2016). Moreover, endocrine regulation of immunity in insects involving some hormones was already described, including JH, which is characterized as an immune suppressor (Nunes et al., 2021). Our result corroborates this influence shown in literature, once coinfected mosquitoes have to deal with a major microorganism invasion.

## 4 Conclusions

Proteomics analysis of *Ae. aegypti* ovaries mono-infected with ZIKV or *Wolbachia* highlighted how those microorganisms interplay almost antagonistic responses considering insect reproducibility and immune response particularities. Our data support that ZIKV induces JH production, probably enhancing insect reproduction capability, while *Wolbachia* seems to harm the same hormone pathway and also egg survival. As for innate insect immunity, *Wolbachia* helps to reinforce *Ae. aegypti* basal features, while ZIKV block it to facilitate infection. During coinfection, *Wolbachia* helps *Ae. aegypti* to prevent virus infection by stimulating ROS production, leading to a Toll-pathway humoral immune response and SOD production to control cell homeostasis. Besides, coinfection showed a potential role between the immune system and reproduction features. Finally, this work should be an important resource for understanding how microorganism infection can influence *Ae. aegypti* immune response and reproducibility, exposing those findings to the insect research community.

## Data Availability Statement

The mass spectrometry proteomics data have been deposited to the ProteomeXchange Consortium via the PRIDE (Perez-Riverol et al., 2022) partner repository with the dataset identifier PXD031728.

## Author Contributions

LR, MM, and JR performed the experiments and data analysis. MM produced paper figures. LR, MM, JR, GD, DO, FN, RM-D-F, and MJ wrote the manuscript. GD, FN, RM-D-F, and MJ provided resources and acquired funding. RM and MJ idealized and coordinated the study. All authors approved the manuscript.

## Funding

The present work was supported by Fundação Carlos Chagas Filho de Amparo à Pesquisa do Estado do Rio de Janeiro (FAPERJ) (Project Temáticos FAPERJ E-26/211.348/2021) and Conselho Nacional de Desenvolvimento Científico e Tecnológico (CNPq).

## Conflict of Interest

The authors declare that the research was conducted in the absence of any commercial or financial relationships that could be construed as a potential conflict of interest.

## Publisher’s Note

All claims expressed in this article are solely those of the authors and do not necessarily represent those of their affiliated organizations, or those of the publisher, the editors and the reviewers. Any product that may be evaluated in this article, or claim that may be made by its manufacturer, is not guaranteed or endorsed by the publisher.

## References

[B1] Ahmad S. Hur S. (2015). Helicases in Antiviral Immunity: Dual Properties as Sensors and Effectors. Trends Biochem. Sci. 40, 576–585. doi: 10.1016/j.tibs.2015.08.001 26410598 PMC4584414

[B2] Aliota M. T. Walker E. C. Yepes A. U. Velez I. D. Christensen B. M. Osorio J. E. (2016). The Wmel Strain of *Wolbachia* Reduces Transmission of Chikungunya Virus in *Aedes Aegypti* . PloS Negl. Trop. Dis. 10, e0004677. doi: 10.1371/journal.pntd.0004677 27124663 PMC4849757

[B3] Allman M. J. Fraser J. E. Ritchie S. A. Joubert D. A. Simmons C. P. Flores H. A. (2020). Wolbachia’s Deleterious Impact on Aedes Aegypti Egg Development: The Potential Role of Nutritional Parasitism. Insects 11, 735. doi: 10.3390/insects11110735 PMC7692218 33120915

[B4] Alomar A. A. Eastmond B. H. Alto B. W. (2021). Juvenile Hormone Analog Enhances Zika Virus Infection in Aedes Aegypti. Sci. Rep. 11, 21062. doi: 10.1038/s41598-021-00432-1 34702871 PMC8548497

[B5] Ant T. H. Herd C. S. Geoghegan V. Hoffmann A. A. Sinkins S. P. (2018). The Wolbachia Strain wAu Provides Highly Efficient Virus Transmission Blocking in Aedes Aegypti. PLoS Pathog. 14, e1006815. doi: 10.1371/journal.ppat.1006815 29370307 PMC5784998

[B6] Ashburner M. Ball C. A. Blake J. A. Botstein D. Butler H. Cherry J. M.. (2000). Gene Ontology: Tool for the Unification of Biology. Nat. Genet. 25, 25–29. doi: 10.1038/75556 10802651 PMC3037419

[B7] Avirutnan P. Hauhart R. E. Marovich M. A. Garred P. Atkinson J. P. Diamond M. S. (2011). Complement-Mediated Neutralization of Dengue Virus Requires Mannose-Binding Lectin. mBio 2, e00276–e00211. doi: 10.1128/mBio.00276-11 22167226 PMC3236064

[B8] Baldaccini M. Pfeffer S. (2021). Untangling the Roles of RNA Helicases in Antiviral Innate Immunity. PLoS Pathog. 17, e1010072. doi: 10.1371/journal.ppat.1010072 34882751 PMC8659333

[B9] Barton L. Sanny J. Dawson E. P. Nouzova M. Noriega F. G. Stadtfeld M.. (2021). Bioactive Isoprenoids Guide Migrating Germ Cells to the Embryonic Gonad. Dev. Biol, 47. doi: 10.1101/2021.09.30.462471

[B10] Blandin S. (2004). Thioester-Containing Proteins and Insect Immunity. Mol. Immunol. 40, 903–908. doi: 10.1016/j.molimm.2003.10.010. BioRxiv pre-print 14698229

[B11] Bose J. Schulte R. D. (2014). Testing GxG Interactions Between Coinfecting Microbial Parasite Genotypes Within Hosts. Front. Genet. 5. doi: 10.3389/fgene.2014.00124 PMC4030146 24860594

[B12] Boyer S. Calvez E. Chouin-Carneiro T. Diallo D. Failloux A.-B. (2018). An Overview of Mosquito Vectors of Zika Virus. Microbes Infect. 20, 646–660. doi: 10.1016/j.micinf.2018.01.006 29481868

[B13] Brinker P. Fontaine M. C. Beukeboom L. W. Falcao Salles J. (2019). Host, Symbionts, and the Microbiome: The Missing Tripartite Interaction. Trends Microbiol. 27, 480–488. doi: 10.1016/j.tim.2019.02.002 30857919

[B14] Buonocore F. Fausto A. M. Della Pelle G. Roncevic T. Gerdol M. Picchietti S. (2021). Attacins: A Promising Class of Insect Antimicrobial Peptides. Antibiotics 10, 212. doi: 10.3390/antibiotics10020212 33672685 PMC7924397

[B15] Campos G. Bandeira A. Sardi S. (2015). Zika Virus Outbreak, Bahia, Brazil. Emerg. Infect. Dis. J. 21, 1885. doi: 10.3201/eid2110.150847 PMC4593454 26401719

[B16] Campos S. S. Fernandes R. S. dos Santos A. A. C. de Miranda R. M. Telleria E. L. Ferreira-de-Brito A.. (2017). Zika Virus can be Venereally Transmitted Between Aedes Aegypti Mosquitoes. Parasites Vectors 10, 605. doi: 10.1186/s13071-017-2543-4 29246237 PMC5731190

[B17] Cao-Lormeau V. M. Roche C. Teissier A. Robin E. Berry A. L. Mallet H. P.. (2014). Zika Virus, French Polynesia, South Pacific 2013 [Letter]. Emerg. Infect. Dis. 20, 1085–1086. doi: 10.3201/eid2006.140138 24856001 PMC4036769

[B18] Chang M.-M. Wang Y.-H. Yang Q.-T. Wang X.-L. Wang M. Raikhel A. S.. (2021). Regulation of Antimicrobial Peptides by Juvenile Hormone and its Receptor, Methoprene-Tolerant, in the Mosquito Aedes Aegypti. Insect Biochem. Mol. Biol. 128, 103509. doi: 10.1016/j.ibmb.2020.103509 33264664

[B19] Charlwood J. D. Tomás E. V. E. Andegiorgish A. K. Mihreteab S. LeClair C. (2018). ‘We Like it Wet’: A Comparison Between Dissection Techniques for the Assessment of Parity in *Anopheles Arabiensis* and Determination of Sac Stage in Mosquitoes Alive or Dead on Collection. PeerJ 6, e5155. doi: 10.7717/peerj.5155 30018854 PMC6044268

[B20] Cheng G. Liu Y. Wang P. Xiao X. (2016). Mosquito Defense Strategies Against Viral Infection. Trends Parasitol. 32, 177–186. doi: 10.1016/j.pt.2015.09.009 26626596 PMC4767563

[B21] Chen X. Sun Y. Zhang T. Shu L. Roepstorff P. Yang F. (2021). Quantitative Proteomics Using Isobaric Labeling: A Practical Guide. Genom. Proteomics Bioinf., 19(5), 689–706. doi: 10.1016/j.gpb.2021.08.012 PMC9170757 35007772

[B22] Chouin-Carneiro T. Vega-Rua A. Vazeille M. Yebakima A. Girod R. Goindin D.. (2016). Differential Susceptibilities of *Aedes Aegypti* and *Aedes Albopictus* From the Americas to Zika Virus. PloS Negl. Trop. Dis. 10, e0004543. doi: 10.1371/journal.pntd.0004543 26938868 PMC4777396

[B23] Christensen S. Pérez Dulzaides R. Hedrick V. E. Momtaz A. J. M. Z. Nakayasu E. S. Paul L. N.. (2016). Wolbachia Endosymbionts Modify Drosophila Ovary Protein Levels in a Context-Dependent Manner. Appl. Environ. Microbiol. 82, 5354–5363. doi: 10.1128/AEM.01255-16 27342560 PMC4988175

[B24] Codeço C. T. Lima A. W. S. Araújo S. C. Lima J. B. P. Maciel-de-Freitas R. Honório N. A.. (2015). Surveillance of Aedes Aegypti: Comparison of House Index With Four Alternative Traps. PloS Negl. Trop. Dis. 9, e0003475. doi: 10.1371/journal.pntd.0003475 25668559 PMC4323346

[B25] Cox J. Mann M. (2011). Quantitative, High-Resolution Proteomics for Data-Driven Systems Biology. Annu. Rev. Biochem. 80, 273–299. doi: 10.1146/annurev-biochem-061308-093216 21548781

[B26] da Silveira I. D. Petersen M. T. Sylvestre G. Garcia G. A. David M. R. Pavan M. G.. (2018). Zika Virus Infection Produces a Reduction on Aedes Aegypti Lifespan But No Effects on Mosquito Fecundity and Oviposition Success. Front. Microbiol. 9. doi: 10.3389/fmicb.2018.03011 PMC6305470 30619118

[B27] De Mandal S. Lin B. Shi M. Li Y. Xu X. Jin F. (2020). iTRAQ-Based Comparative Proteomic Analysis of Larval Midgut From the Beet Armyworm, *Spodoptera Exigua* (Hübner) (Lepidoptera: Noctuidae) Challenged With the Entomopathogenic Bacteria *Serratia Marcescens* . Front. Physiol. 11. doi: 10.3389/fphys.2020.00442 PMC7227483 32457652

[B28] Dick G. W. A. Kitchen S. F. Haddow A. J. (1952). Zika Virus (I). Isolations and Serological Specificity. Trans. R. Soc. Trop. Med. Hygiene. 46, 509–520. doi: 10.1016/0035-9203(52)90042-4 12995440

[B29] Dutra H. L. C. dos Santos L. M. B. Caragata E. P. Silva J. B. L. Villela D. A. M. Maciel-de-Freitas R.. (2015). From Lab to Field: The Influence of Urban Landscapes on the Invasive Potential of Wolbachia in Brazilian Aedes Aegypti Mosquitoes. PloS Negl. Trop. Dis. 9, e0003689. doi: 10.1371/journal.pntd.0003689 25905888 PMC4408005

[B30] Dutra H. L. C. Rocha M. N. Dias F. B. S. Mansur S. B. Caragata E. P. Moreira L. A. (2016). *Wolbachia* Blocks Currently Circulating Zika Virus Isolates in Brazilian *Aedes Aegypti* Mosquitoes. Cell Host Microbe 19, 771–774. doi: 10.1016/j.chom.2016.04.021 27156023 PMC4906366

[B31] Engelmann F. (1979). “Insect Vitellogenin: Identification, Biosynthesis, and Role in Vitellogenesis,” in Advances in Insect Physiology (Los Angeles, USA:Elsevier), 49–108. doi: 10.1016/S0065-2806(08)60051-X

[B32] Epelboin Y. Talaga S. Epelboin L. Dusfour I. (2017). Zika Virus: An Updated Review of Competent or Naturally Infected Mosquitoes. PloS Negl. Trop. Dis. 11, e0005933. doi: 10.1371/journal.pntd.0005933 29145400 PMC5690600

[B33] Farnesi L. C. Belinato T. A. Gesto J. S. M. Martins A. J. Bruno R. V. Moreira L. A. (2019). Embryonic Development and Egg Viability of Wmel-Infected Aedes Aegypti. Parasites Vectors 12, 211. doi: 10.1186/s13071-019-3474-z 31060581 PMC6503365

[B34] Fernandes R. S. Campos S. S. Ferreira-de-Brito A. Miranda R. M. de, Silva K.A.B.da Castro M.G.de. (2016). *Culex Quinquefasciatus* From Rio De Janeiro Is Not Competent to Transmit the Local Zika Virus. PloS Negl. Trop. Dis. 10, e0004993. doi: 10.1371/journal.pntd.0004993 27598421 PMC5012671

[B35] Ferreira-de-Brito A. Ribeiro I. P. Miranda R.M.de Fernandes R. S. Campos S. S. Silva K.A.B.da. (2016). First Detection of Natural Infection of *Aedes Aegypti* With Zika Virus in Brazil and Throughout South America. Memórias. do Instituto. Oswaldo. Cruz. 111, 655–658. doi: 10.1590/0074-02760160332 27706382 PMC5066335

[B36] Ford S. A. Albert I. Allen S. L. Chenoweth S. F. Jones M. Koh C.. (2020). Artificial Selection Finds New Hypotheses for the Mechanism of *Wolbachia-*Mediated Dengue Blocking in Mosquitoes. Front. Microbiol. 11. doi: 10.3389/fmicb.2020.01456 PMC7358395 32733407

[B37] Fuchs A. Pinto A. K. Schwaeble W. J. Diamond M. S. (2011). The Lectin Pathway of Complement Activation Contributes to Protection From West Nile Virus Infection. Virology 412, 101–109. doi: 10.1016/j.virol.2011.01.003 21269656 PMC3057364

[B38] Garcia G. de A. Sylvestre G. Aguiar R. Costa G.B.da Martins A. J.. (2019). Matching the Genetics of Released and Local *Aedes Aegypti* Populations is Critical to Assure *Wolbachia* Invasion. PloS Negl. Trop. Dis. 13, e0007023. doi: 10.1371/journal.pntd.0007023 30620733 PMC6338382

[B39] Garcia G. A. Hoffmann A. A. Maciel-de-Freitas R. Villela D. A. M. (2020). Aedes Aegypti Insecticide Resistance Underlies the Success (and Failure) of Wolbachia Population Replacement. Sci. Rep. 10, 63. doi: 10.1038/s41598-019-56766-4 31919396 PMC6952458

[B40] García-Robles I. De Loma J. Capilla M. Roger I. Boix-Montesinos P. Carrión P.. (2020). Proteomic Insights Into the Immune Response of the Colorado Potato Beetle Larvae Challenged With *Bacillus Thuringiensis* . Dev. Comp. Immunol. 104, 103525. doi: 10.1016/j.dci.2019.103525 31655128

[B41] Geiser D. Li W. Pham D. Q. D. Wysocki V. H. Winzerling J. J.. (2022). Shotgun and TMT-Labeled Proteomic Analysis of the Ovarian Proteins of an Insect Vector, Aedes aegypti (Diptera: Culicidae). J Insect Sci 22, 7. doi: 10.1093/jisesa/ieac018 PMC8932505 35303100

[B42] Geng T. Lu F. Wu H. Wang Y. Lou D. Tu N.. (2021). C-Type Lectin 5, a Novel Pattern Recognition Receptor for the JAK/STAT Signaling Pathway in Bombyx Mori. J. Invertebrate. Pathol. 179, 107473. doi: 10.1016/j.jip.2020.107473 32946913

[B43] Gesto J. S. M. Ribeiro G. S. Rocha M. N. Dias F. B. S. Peixoto J. Carvalho F. D.. (2021). Reduced Competence to Arboviruses Following the Sustainable Invasion of Wolbachia Into Native Aedes Aegypti From Southeastern Brazil. Sci. Rep. 11, 10039. doi: 10.1038/s41598-021-89409-8 33976301 PMC8113270

[B44] Gestuveo R. J. Royle J. Donald C. L. Lamont D. J. Hutchinson E. C. Merits A.. (2021). Analysis of Zika Virus Capsid-Aedes Aegypti Mosquito Interactome Reveals Pro-Viral Host Factors Critical for Establishing Infection. Nat. Commun. 12, 2766. doi: 10.1038/s41467-021-22966-8 33986255 PMC8119459

[B45] Giraldo-Calderón G. I. Emrich S. J. MacCallum R. M. Maslen G. Dialynas E. Topalis P.. (2014). VectorBase: An Updated Bioinformatics Resource for Invertebrate Vectors and Other Organisms Related With Human Diseases. Nucleic Acids Res. 43, 707–713. doi: 10.1093/nar/gku1117 PMC4383932 25510499

[B46] Gloria-Soria A. Chiodo T. G. Powell J. R. (2018). Lack of Evidence for Natural Wolbachia Infections in Aedes Aegypti (Diptera: Culicidae). J. Med. Entomol. 55(5), 1354–1356. doi: 10.1093/jme/tjy084 29901734 PMC6113644

[B47] Göertz G. P. van Bree J. W. M. Hiralal A. Fernhout B. M. Steffens C. Boeren S.. (2019). Subgenomic Flavivirus RNA Binds the Mosquito DEAD/H-Box Helicase ME31B and Determines Zika Virus Transmission by *Aedes Aegypti* . Proc. Natl. Acad. Sci. U.S.A. 116, 19136–19144. doi: 10.1073/pnas.1905617116 31488709 PMC6754610

[B48] González M. A. Pavan M. G. Fernandes R. S. Busquets N. David M. R. Lourenço-Oliveira R.. (2019). Limited Risk of Zika Virus Transmission by Five Aedes Albopictus Populations From Spain. Parasites Vectors 12, 150. doi: 10.1186/s13071-019-3359-1 30922370 PMC6440144

[B49] Guo Y. Hoffmann A. A. Xu X.-Q. Mo P.-W. Huang H.-J. Gong J.-T.. (2018). Vertical Transmission of Wolbachia Is Associated With Host Vitellogenin in Laodelphax Striatellus. Front. Microbiol. 9. doi: 10.3389/fmicb.2018.02016 PMC6127624 30233514

[B50] Indriani C. Tantowijoyo W. Rancès E. Andari B. Prabowo E. Yusdi D.. (2020). Reduced Dengue Incidence Following Deployments of *Wolbachia-*Infected Aedes Aegypti in Yogyakarta, Indonesia: A Quasi-Experimental Trial Using Controlled Interrupted Time Series Analysis. Gates. Open Res. 4, 50. doi: 10.12688/gatesopenres.13122.1 32803130 PMC7403856

[B51] Jindra M. Bellés X. Shinoda T. (2015). Molecular Basis of Juvenile Hormone Signaling. Curr. Opin. Insect Sci. 11, 39–46. doi: 10.1016/j.cois.2015.08.004 28285758

[B52] Kaur R. Shropshire J. D. Cross K. L. Leigh B. Mansueto A. J. Stewart V.. (2021). Living in the Endosymbiotic World of Wolbachia: A Centennial Review. Cell Host Microbe 29, 879–893. doi: 10.1016/j.chom.2021.03.006 33945798 PMC8192442

[B53] King J. G. Souto-Maior C. Sartori L. M. Maciel-de-Freitas R. Gomes M. G. M. (2018). Variation in Wolbachia Effects on Aedes Mosquitoes as a Determinant of Invasiveness and Vectorial Capacity. Nat. Commun. 9, 1483. doi: 10.1038/s41467-018-03981-8 29662096 PMC5902584

[B54] Lai Z. Zhou T. Zhou J. Liu S. Xu Y. Gu J.. (2020). Vertical Transmission of Zika Virus in Aedes Albopictus. PloS Negl. Trop. Dis. 14, e0008776. doi: 10.1371/journal.pntd.0008776 33057411 PMC7671534

[B55] Lau M.-J. Ross P. A. Hoffmann A. A. (2021). Infertility and Fecundity Loss of Wolbachia-Infected Aedes Aegypti Hatched From Quiescent Eggs is Expected to Alter Invasion Dynamics. PloS Negl. Trop. Dis. 15, e0009179. doi: 10.1371/journal.pntd.0009179 33591971 PMC7909672

[B56] Leitner M. Bishop C. Asgari S.. (2021). Transcriptional Response of Wolbachia to Dengue Virus Infection in Cells of the Mosquito Aedes aegypti. mSphere 6 ,e00433–21. doi: 10.1128/mSphere.00433-21 PMC8265661 34190587

[B57] LePage D. P. Metcalf J. A. Bordenstein S. R. On J. Perlmutter J. I. Shropshire J. D.. (2017). Prophage WO Genes Recapitulate and Enhance Wolbachia-Induced Cytoplasmic Incompatibility. Nature 543, 243–247. doi: 10.1038/nature21391 28241146 PMC5358093

[B58] Li C. Guo X. Deng Y. Xing D. Sun A. Liu Q.. (2017). Vector Competence and Transovarial Transmission of Two *Aedes Aegypti* Strains to Zika Virus. Emerg. Microbes Infect. 6, 1–7. doi: 10.1038/emi.2017.8 PMC5457675 28442754

[B59] Li Q. Meng Q.-W. Lü F.-G. Guo W.-C. Li G.-Q. (2016). Identification of Ten Mevalonate Enzyme-Encoding Genes and Their Expression in Response to Juvenile Hormone Levels in Leptinotarsa Decemlineata (Say). Gene 584, 136–147. doi: 10.1016/j.gene.2016.02.023 26899871

[B60] Lomate P. R. Sangole K. P. Sunkar R. Hivrale V. K. (2015). Superoxide Dismutase Activities in the Midgut of Helicoverpa Armigera Larvae: Identification and Biochemical Properties of a Manganese Superoxide Dismutase. OAIP 5, 13–20. doi: 10.2147/OAIP.S84053

[B61] Mancini M. V. Herd C. S. Ant T. H. Murdochy S. M. Sinkins S. P. (2020). Wolbachia Strain wAu Efficiently Blocks Arbovirus Transmission in Aedes Albopictus. PloS Negl. Trop. Dis. 14, e0007926. doi: 10.1371/journal.pntd.0007926 32155143 PMC7083328

[B62] Manuel M. Missé D. Pompon J. (2020). Highly Efficient Vertical Transmission for Zika Virus in Aedes Aegypti After Long Extrinsic Incubation Time. Pathogens 9, 366. doi: 10.3390/pathogens9050366 PMC7281418 32403319

[B63] Ma Z. Qu B. Yao L. Gao Z. Zhang S. (2020). Identification and Functional Characterization of Ribosomal Protein S23 as a New Member of Antimicrobial Protein. Dev. Comp. Immunol. 110, 103730. doi: 10.1016/j.dci.2020.103730 32423862

[B64] Martins M. Ramos L. F. C. Murillo J. R. Torres A. de Carvalho S. S. Domont G. B.. (2021). Comprehensive Quantitative Proteome Analysis of Aedes Aegypti Identifies Proteins and Pathways Involved in Wolbachia Pipientis and Zika Virus Interference Phenomenon. Front. Physiol. 12. doi: 10.3389/fphys.2021.642237 PMC7947915 33716790

[B65] Mayoral J. G. Nouzova M. Navare A. Noriega F. G. (2009). NADP ^+^ -Dependent Farnesol Dehydrogenase, a *Corpora Allata* Enzyme Involved in Juvenile Hormone Synthesis. PNAS 106, 21091–21096. doi: 10.1073/pnas.0909938106 19940247 PMC2795498

[B66] McMeniman C. J. Hughes G. L. O’Neill S. L. (2011). A Wolbachia Symbiont in Aedes Aegypti Disrupts Mosquito Egg Development to a Greater Extent When Mosquitoes Feed on Nonhuman Versus Human Blood. jnl. Med. entom. 48, 76–84. doi: 10.1603/ME09188 21337952

[B67] Moreira L. A. Iturbe-Ormaetxe I. Jeffery J. A. Lu G. Pyke A. T. Hedges L. M.. (2009). A *Wolbachia* Symbiont in *Aedes Aegypti* Limits Infection With Dengue, Chikungunya, and Plasmodium. Cell 139, 1268–1278. doi: 10.1016/j.cell.2009.11.042 20064373

[B68] Mukherjee D. Das S. Begum F. Mal S. Ray U. (2019). The Mosquito Immune System and the Life of Dengue Virus: What We Know and Do Not Know. Pathogens 8, 77. doi: 10.3390/pathogens8020077 PMC6631187 31200426

[B69] Nag D. K. Payne A. F. Dieme C. Ciota A. T. Kramer L. D. (2021). Zika Virus Infects Aedes Aegypti Ovaries. Virology 561, 58–64. doi: 10.1016/j.virol.2021.06.002 34147955 PMC10117528

[B70] Nazni W. A. Hoffmann A. A. NoorAfizah A. Cheong Y. L. Mancini M. V. Golding N.. (2019). Establishment of *Wolbachia* Strain Walbb in Malaysian Populations of *Aedes Aegypti* for Dengue Control. Curr. Biol. 29, 4241–4248.e5. doi: 10.1016/j.cub.2019.11.007 31761702 PMC6926472

[B71] Noh M. Y. Kim S. H. Gorman M. J. Kramer K. J. Muthukrishnan S. Arakane Y. (2020). Yellow-G and Yellow-G2 Proteins are Required for Egg Desiccation Resistance and Temporal Pigmentation in the Asian Tiger Mosquito, Aedes Albopictus. Insect Biochem. Mol. Biol. 122, 103386. doi: 10.1016/j.ibmb.2020.103386 32315743

[B72] Noh M. Y. Kramer K. J. Muthukrishnan S. Beeman R. W. Kanost M. R. Arakane Y. (2015). Loss of Function of the Yellow-E Gene Causes Dehydration-Induced Mortality of Adult Tribolium Castaneum. Dev. Biol. 399, 315–324. doi: 10.1016/j.ydbio.2015.01.009 25614237

[B73] Noriega F. G. (2014). Juvenile Hormone Biosynthesis in Insects: What Is New, What Do We Know, and What Questions Remain? Int. Scholarly. Res. Notices. 2014, 1–16. doi: 10.1155/2014/967361 PMC4897325 27382622

[B74] Nouzova M. Edwards M. J. Mayoral J. G. Noriega F. G. (2011). A Coordinated Expression of Biosynthetic Enzymes Controls the Flux of Juvenile Hormone Precursors in the Corpora Allata of Mosquitoes. Insect Biochem. Mol. Biol. 41, 660–669. doi: 10.1016/j.ibmb.2011.04.008 21554954 PMC3129432

[B75] Nunes C. Sucena É. Koyama T. (2021). Endocrine Regulation of Immunity in Insects. FEBS J. 288, 3928–3947. doi: 10.1111/febs.15581 33021015

[B76] Ogunlade S. T. Meehan M. T. Adekunle A. I. Rojas D. P. Adegboye O. A. McBryde E. S. (2021). A Review: Aedes-Borne Arboviral Infections, Controls and Wolbachia-Based Strategies. Vaccines 9, 32. doi: 10.3390/vaccines9010032 33435566 PMC7827552

[B77] Pan X. Zhou G. Wu J. Bian G. Lu P. Raikhel A. S.. (2012). *Wolbachia* Induces Reactive Oxygen Species (ROS)-Dependent Activation of the Toll Pathway to Control Dengue Virus in the Mosquito Aedes Aegypti. PNAS 109, E23–E31. doi: 10.1073/pnas.1116932108 22123956 PMC3252928

[B78] Park D. H. Choi J. Y. Lee S.-H. Kim J. H. Park M. G. Kim J. Y.. (2020). Mosquito Larvicidal Activities of Farnesol and Farnesyl Acetate *via* Regulation of Juvenile Hormone Receptor Complex Formation in Aedes Mosquito. J. Asia-Pacific. Entomol. 23, 689–693. doi: 10.1016/j.aspen.2020.05.006

[B79] Park S.-Y. Nair P. M. G. Choi J. (2012). Characterization and Expression of Superoxide Dismutase Genes in Chironomus Riparius (Diptera, Chironomidae) Larvae as a Potential Biomarker of Ecotoxicity. Comp. Biochem. Physiol. Part C.: Toxicol. Pharmacol. 156, 187–194. doi: 10.1016/j.cbpc.2012.06.003 22710426

[B80] Perez-Riverol Y. Bai J. Bandla C. Hewapathirana S. García-Seisdedos D. Kamatchinathan S.. (2022). The PRIDE Database Resources in 2022: A Hub for Mass Spectrometry-Based Proteomics Evidences. Nucleic Acids Res. 50 (D1), D543–D552. doi: 10.1093/nar/gkab1038 34723319 PMC8728295

[B81] Petersen M. T. Silveira I.D.da Tátila-Ferreira A. David M. R. Chouin-Carneiro T. Wouwer L.V.d.. (2018). The Impact of the Age of First Blood Meal and Zika Virus Infection on Aedes Aegypti Egg Production and Longevity. PloS One 13, e0200766. doi: 10.1371/journal.pone.0200766 30048481 PMC6062029

[B82] Pickett B. E. Sadat E. L. Zhang Y. Noronha J. M. Squires R. B. Hunt V.. (2012). ViPR: An Open Bioinformatics Database and Analysis Resource for Virology Research. Nucleic Acids Res. 40, D593–D598. doi: 10.1093/nar/gkr859 22006842 PMC3245011

[B83] Pietri J. E. DeBruhl H. Sullivan W. (2016). The Rich Somatic Life of *Wolbachia* . MicrobiologyOpen 5, 923–936. doi: 10.1002/mbo3.390 27461737 PMC5221451

[B84] Pimentel A. C. Cesar C. S. Martins M. Cogni R. (2021). The Antiviral Effects of the Symbiont Bacteria Wolbachia in Insects. Front. Immunol. 11. doi: 10.3389/fimmu.2020.626329 PMC7878553 33584729

[B85] Rocha-Santos C. Dutra A. C. V. P. L. Fróes Santos R. Cupolillo C. D. L. S. de Melo Rodovalho C. Bellinato D. F.. (2021). Effect of Larval Food Availability on Adult *Aedes Aegypti* (Diptera: Culicidae) Fitness and Susceptibility to Zika Infection. J. Med. Entomol. 58, 535–547. doi: 10.1093/jme/tjaa249 33219384

[B86] Ross P. A. Endersby N. M. Yeap H. L. Hoffmann A. A. (2014). Larval Competition Extends Developmental Time and Decreases Adult Size of Wmelpop Wolbachia-Infected Aedes Aegypti. Am J Trop Med Hygiene 91, 198–205. doi: 10.4269/ajtmh.13-0576 PMC4080562 24732463

[B87] Ryan P. A. Turley A. P. Wilson G. Hurst T. P. Retzki K. Brown-Kenyon J.. (2020). Establishment of Wmel *Wolbachia* in *Aedes Aegypti* Mosquitoes and Reduction of Local Dengue Transmission in Cairns and Surrounding Locations in Northern Queensland, Australia. Gates. Open Res. 3, 1547. doi: 10.12688/gatesopenres.13061.2 31667465 PMC6801363

[B88] Sá-Guimarães T. E. Salles T. S. Santos C. R. Moreira M. F. Souza W. Caldas L. A. (2021). Route of Zika Virus Infection in *Aedes Aegypti* by Transmission Electron Microscopy. BMC Microbiol. 21, 300. doi: 10.1186/s12866-021-02366-0 34717555 PMC8557066

[B89] Santos C. G. Humann F. C. Hartfelder K. (2019). Juvenile Hormone Signaling in Insect Oogenesis. Curr. Opin. Insect Sci. 31, 43–48. doi: 10.1016/j.cois.2018.07.010 31109672

[B90] Schneider S. M. Lee B. H. Nicola A. V. (2021). Viral Entry and the Ubiquitin-Proteasome System. Cell. Microbiol. 23, 1–12. doi: 10.1111/cmi.13276 33037857

[B91] Schubert O. T. Röst H. L. Collins B. C. Rosenberger G. Aebersold R. (2017). Quantitative Proteomics: Challenges and Opportunities in Basic and Applied Research. Nat. Protoc. 12, 1289–1294. doi: 10.1038/nprot.2017.040 28569762

[B92] Schwenke R. A. Lazzaro B. P. Wolfner M. F. (2016). Reproduction–Immunity Trade-Offs in Insects. Annu. Rev. Entomol. 61, 239–256. doi: 10.1146/annurev-ento-010715-023924 26667271 PMC5231921

[B93] Serteyn L. Ponnet L. Saive M. Fauconnier M.-L. Francis F. (2020). Changes of Feeding Behavior and Salivary Proteome of Brown Marmorated Stink Bug When Exposed to Insect-Induced Plant Defenses. Arthropod-Plant. Interact. 14, 101–112. doi: 10.1007/s11829-019-09718-8

[B94] Sinha A. Li Z. Sun L. Carlow C. K. S. (2019). Complete Genome Sequence of the Wolbachia w AlbB Endosymbiont of Aedes albopictus. Genome Biol. Evol. 11, 706–720. doi: 10.1093/gbe/evz025 30715337 PMC6414309

[B95] Shapiro D. Taylor J. M. (1982). Steroid Hormone Regulation of Vitellogenin Gene Expressio. Crit. Rev. Biochem. 12, 187–203. doi: 10.3109/10409238209108706 6123408

[B96] Shi Z.-K. Wen D. Chang M.-M. Sun X.-M. Wang Y.-H. Cheng C.-H.. (2021). Juvenile Hormone-Sensitive Ribosomal Activity Enhances Viral Replication in Aedes Aegypti. mSystems 6, 1–16. doi: 10.1128/mSystems.01190-20 PMC8269256 34061577

[B97] Shokal U. Eleftherianos I. (2017). Evolution and Function of Thioester-Containing Proteins and the Complement System in the Innate Immune Response. Front. Immunol. 8. doi: 10.3389/fimmu.2017.00759 PMC5489563 28706521

[B98] Sim C. Denlinger D. L. (2011). Catalase and Superoxide Dismutase-2 Enhance Survival and Protect Ovaries During Overwintering Diapause in the Mosquito Culex Pipiens. J. Insect Physiol. 57, 628–634. doi: 10.1016/j.jinsphys.2011.01.012 21277308 PMC3104096

[B99] Smith L. Kelleher N. The Consortium for Top Down Proteomic (2013). Proteoform: A Single Term Describing Protein Complexity. Nat. Methods 10, 186–187. doi: 10.1038/nmeth.2369 23443629 PMC4114032

[B100] Su C. Tang Y. Zheng C. (2022). DExD/H-Box Helicases: Multifunctional Regulators in Antiviral Innate Immunity. Cell. Mol. Life Sci. 79, 2. doi: 10.1007/s00018-021-04072-6 PMC8671602 34910251

[B101] Takahashi K. Ip W. E. Michelow I. C. Ezekowitz R. A. B. (2006). The Mannose-Binding Lectin: A Prototypic Pattern Recognition Molecule. Curr. Opin. Immunol. 18, 16–23. doi: 10.1016/j.coi.2005.11.014 16368230 PMC7126801

[B102] Taschuk F. Cherry S. (2020). DEAD-Box Helicases: Sensors, Regulators, and Effectors for Antiviral Defense. Viruses 12, 181. doi: 10.3390/v12020181 PMC7077277 32033386

[B103] Thangamani S. Huang J. Hart C. E. Guzman H. Tesh R. B. (2016). Vertical Transmission of Zika Virus in Aedes Aegypti Mosquitoes. Am. J. Trop. Med. Hygiene. 95, 1169–1173. doi: 10.4269/ajtmh.16-0448 PMC5094235 27573623

[B104] Tsang S. S. K. Law S. T. S. Li C. Qu Z. Bendena W. G. Tobe S. S.. (2020). Diversity of Insect Sesquiterpenoid Regulation. Front. Genet. 11. doi: 10.3389/fgene.2020.01027 PMC7511761 33133135

[B105] Tyanova S. Temu T. Sinitcyn P. Carlson A. Hein M. Y. Geiger T.. (2016). The Perseus Computational Platform for Comprehensive Analysis of (Prote)omics Data. Nature Methods 13, 731–740. doi: 10.1038/nmeth.3901 27348712

[B106] Utarini A. Indriani C. Ahmad R. A. Tantowijoyo W. Arguni E. Ansari M. R.. (2021). Efficacy of Wolbachia-Infected Mosquito Deployments for the Control of Dengue. N. Engl. J. Med. 384, 2177–2186. doi: 10.1056/NEJMoa2030243 34107180 PMC8103655

[B107] Wang W. Yang R.-R. Peng L.-Y. Zhang L. Yao Y.-L. Bao Y.-Y. (2021). Proteolytic Activity of the Proteasome is Required for Female Insect Reproduction. Open Biol. 11, rsob.200251, 200251. doi: 10.1098/rsob.200251 PMC8061697 33622101

[B108] Waterhouse R. M. Povelones M. Christophides G. K. (2010). Sequence-Structure-Function Relations of the Mosquito Leucine-Rich Repeat Immune Proteins. BMC Genomics 11, 531. doi: 10.1186/1471-2164-11-531 20920294 PMC3020904

[B109] Wigglesworth V. B. (1934). The Physiology of Ecdysis in Rhodnius Pro- Lixus (Hemiptera). II. Factors Controlling Moulting and “Metamorphosis”. London School of Hygiene and Tropical Medicine: London, UK 33.

[B110] Wong Z. S. Hedges L. M. Brownlie J. C. Johnson K. N. (2011). Wolbachia-Mediated Antibacterial Protection and Immune Gene Regulation in Drosophila. PLoS One 6, e25430. doi: 10.1371/journal.pone.0025430 21980455 PMC3183045

[B111] Wong Z. S. Hedges L. M. Brownlie J. C. Johnson K. N. (2011). Wolbachia-Mediated Antibacterial Protection and Immune Gene Regulation in Drosophila. PLoS One 6, e25430. doi: 10.1371/journal.pone.0025430 21980455 PMC3183045

[B112] Wu Q. Patočka J. Kuča K. (2018). Insect Antimicrobial Peptides, a Mini Review. Toxins 10, 461. doi: 10.3390/toxins10110461 PMC6267271 30413046

[B113] Wu Z. Yang L. He Q. Zhou S. (2021). Regulatory Mechanisms of Vitellogenesis in Insects. Front. Cell Dev. Biol. 8. doi: 10.3389/fcell.2020.593613 PMC7901893 33634094

[B114] Xiao X. Liu Y. Zhang X. Wang J. Li Z. Pang X.. (2014). Complement-Related Proteins Control the Flavivirus Infection of Aedes Aegypti by Inducing Antimicrobial Peptides. PLoS Pathog. 10, e1004027. doi: 10.1371/journal.ppat.1004027 24722701 PMC3983052

[B115] Xia X. You M. Rao X.-J. Yu X.-Q. (2018). Insect C-Type Lectins in Innate Immunity. Dev. Comp. Immunol. 83, 70–79. doi: 10.1016/j.dci.2017.11.020 29198776

[B116] Xi Z. Ramirez J. L. Dimopoulos G. (2008). The Aedes Aegypti Toll Pathway Controls Dengue Virus Infection. PLoS Pathog. 4, e1000098. doi: 10.1371/journal.ppat.1000098 18604274 PMC2435278

[B117] Ye Y. H. Woolfit M. Rancès E. O’Neill S. L. McGraw E. A. (2013). Wolbachia-Associated Bacterial Protection in the Mosquito Aedes Aegypti. PloS Negl. Trop. Dis. 7, e2362. doi: 10.1371/journal.pntd.0002362 23951381 PMC3738474

[B118] Zhang R. Zhu Y. Pang X. Xiao X. Zhang R. Cheng G. (2017). Regulation of Antimicrobial Peptides in Aedes Aegypti Aag2 Cells. Front. Cell. Infect. Microbiol. 7. doi: 10.3389/fcimb.2017.00022 PMC5291090 28217557

[B119] Zhao L. Alto B. Shin D. (2019). Transcriptional Profile of Aedes Aegypti Leucine-Rich Repeat Proteins in Response to Zika and Chikungunya Viruses. IJMS 20, 615. doi: 10.3390/ijms20030615 PMC6386990 30708982

[B120] Zifruddin A.-N. Mohamad-Khalid K.-A. Suhaimi S.-A. Mohamed-Hussein Z.-A. Hassan M. (2021). Molecular Characterization and Enzyme Inhibition Studies of NADP+- Farnesol Dehydrogenase From Diamondback Moth, *Plutella Xylostella* (Lepidoptera: Plutellidae). Bioscience. Biotechn. Biochem. 85, 1628–1638. doi: 10.1093/bbb/zbab072 33890631

[B121] Zimler R. A. Yee D. A. Alto B. W. (2021). Transmission Potential of Zika Virus by *Aedes Aegypti* (Diptera: Culicidae) and *Ae. Mediovittatus* (Diptera: Culicidae) Populations From Puerto Rico. J. Med. Entomol. 58, 1405–1411. doi: 10.1093/jme/tjaa286 33554254 PMC8122233

[B122] Zug R. Hammerstein P. (2015). *Wolbachia* and the Insect Immune System: What Reactive Oxygen Species can Tell Us About the Mechanisms of *Wolbachia*–host Interactions. Front. Microbiol. 6. doi: 10.3389/fmicb.2015.01201 PMC4621438 26579107

