# Supplementary figures and images for: Interspecies Isobaric Labeling-Based Quantitative Proteomics Reveals Protein Changes in the Ovary of Aedes aegypti Coinfected With ZIKV and Wolbachia
Source: Front Cell Infect Microbiol. 2022 Jul 7;12:900608. doi: 10.3389/fcimb.2022.900608 (PMC9302590; doi:10.3389/fcimb.2022.900608)

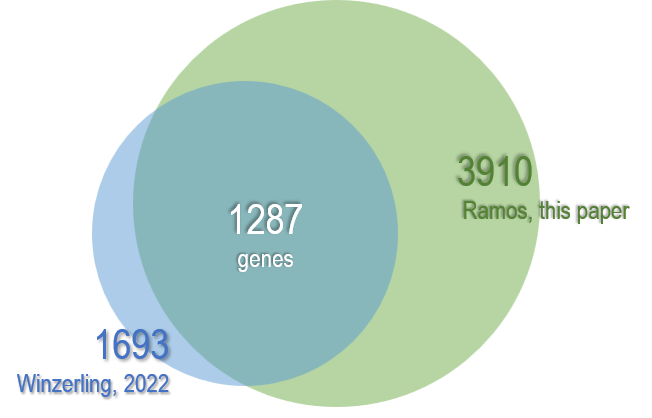

Supplement: Supplementary file 1 [file Image_1.tiff]

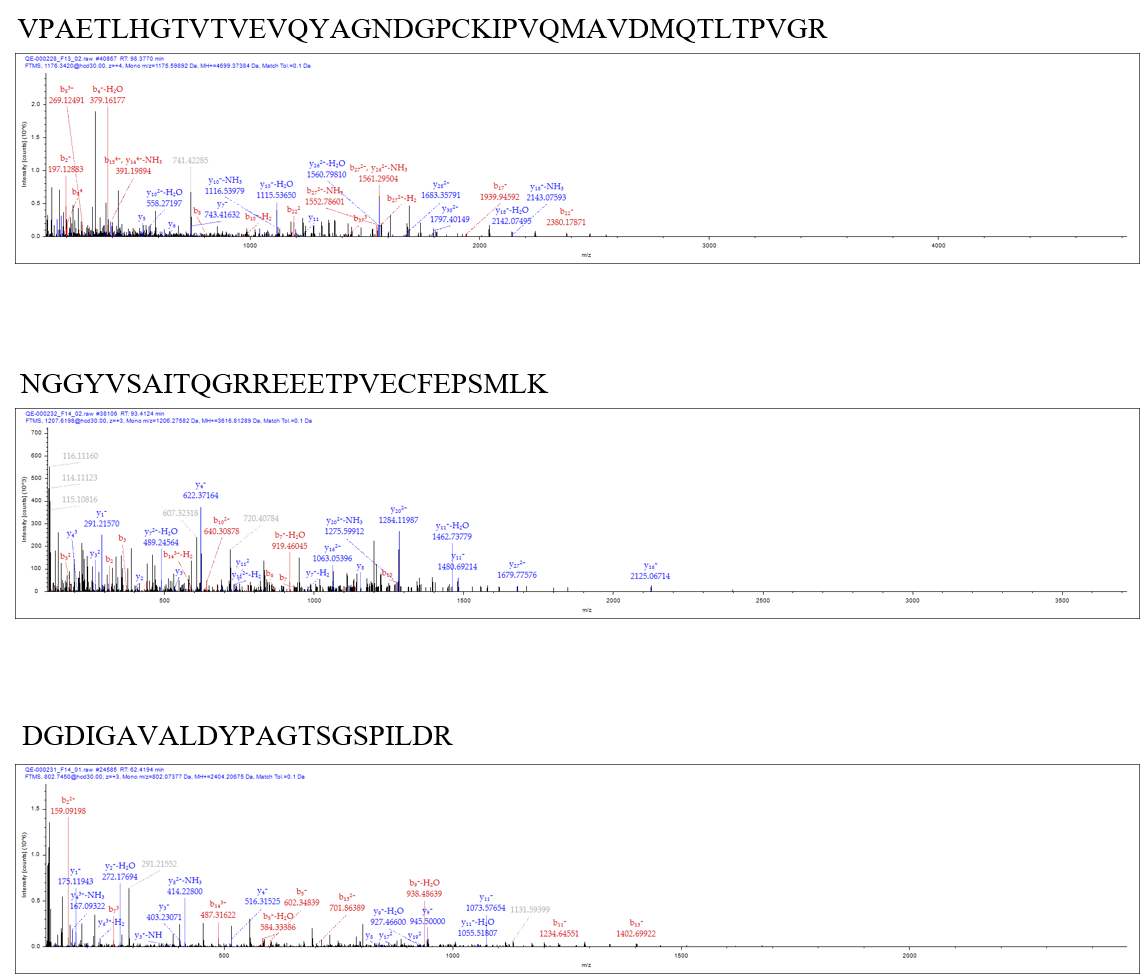

Supplement: Supplementary file 2 [file Image_2.tiff]
